# The increased presence of repetitive motifs in the KDDR-plus recombinant protein, a kinesin-derived antigen from *Leishmania infantum*, improves the diagnostic performance of serological tests for human and canine visceral leishmaniasis

**DOI:** 10.1371/journal.pntd.0009759

**Published:** 2021-09-17

**Authors:** Williane Fernanda Siqueira, Agostinho Gonçalves Viana, João Luís Reis Cunha, Leticia Mansur Rosa, Lilian Lacerda Bueno, Daniella Castanheira Bartholomeu, Mariana Santos Cardoso, Ricardo Toshio Fujiwara

**Affiliations:** 1 Programa de Pós-graduação em Ciências da Saúde: Infectologia e Medicina Tropical, Faculdade de Medicina, Universidade Federal de Minas Gerais, Belo Horizonte, Minas Gerais, Brazil; 2 Departamento de Parasitologia, Instituto de Ciências Biológicas, Universidade Federal de Minas Gerais, Belo Horizonte, Minas Gerais, Brazil; Centro de Pesquisa Gonçalo Moniz-FIOCRUZ/BA, BRAZIL

## Abstract

Visceral leishmaniasis (VL) is caused by protozoa belonging to the *Leishmania donovani* complex and is considered the most serious and fatal form among the different types of leishmaniasis, if not early diagnosed and treated. Among the measures of disease control stand out the management of infected dogs and the early diagnosis and appropriate treatment of human cases. Several antigens have been characterized for use in the VL diagnosis, among them are the recombinant kinesin-derived antigens from *L*. *infantum*, as rK39 and rKDDR. The main difference between these antigens is the size of the non-repetitive kinesin region and the number of repetitions of the 39 amino acid degenerate motif (6.5 and 8.5 repeats in rK39 and rKDDR, respectively). This repetitive region has a high antigenicity score. To evaluate the effect of increasing the number of repeats on diagnostic performance, we designed the rKDDR-plus antigen, containing 15.3 repeats of the 39 amino acid degenerate motif, besides the absence of the non-repetitive portion from *L*. *infantum* kinesin. Its performance was evaluated by enzyme-linked immunosorbent assay (ELISA) and rapid immunochromatographic test (ICT), and compared with the kinesin-derived antigens (rKDDR and rK39). In ELISA with human sera, all recombinant antigens had a sensitivity of 98%, whereas the specificity for rKDDR-plus, rKDDR and rK39 was 100%, 96% and 71%, respectively. When evaluated canine sera, the ELISA sensitivity was 97% for all antigens, and the specificity for rKDDR-plus, rKDDR and rK39 was 98%, 91% and 83%, respectively. Evaluation of the ICT/rKDDR-plus, using human sera, showed greater diagnostic sensitivity (90%) and specificity (100%), when compared to the IT LEISH (79% and 98%, respectively), which is based on the rK39 antigen. These results suggest that the increased presence of repetitive motifs in the rKDDR-plus protein improves the diagnostic performance of serological tests by increasing the specificity and accuracy of the diagnosis.

## Author summary

Visceral Leishmaniasis (VL) is a neglected tropical disease caused by protozoa *Leishmania infantum* and *L*. *donovani*. This illness affects millions of people in various regions of the world and can be fatal if not early treated. In the Americas and Southern Europe, the VL is a zoonotic disease, being the dog its main reservoir. The early and correct diagnosis of dogs and humans infected with *Leishmania* is essential for the efficient disease control. However, current diagnostic methods have limitations, as they are not sufficiently sensitive and/or specific, and may not be able to identify all cases of VL or exhibit cross-reactions with other infections. We produced a recombinant protein, rKDDR-plus, constituted only by highly repetitive sequences kinesin-derived from *L*. *infantum*, and compare its performance with other kinesin-derived antigens (rK39 and rKDDR), but that have less repetitive sequences. The rKDDR-plus protein exhibited the best performance among the studied antigens, presenting a greater specificity in the VL diagnosis, both in the ELISA and rapid test platforms. Therefore, this antigen can contribute to the control of leishmaniasis in humans and dogs, through a diagnostic test with high specificity, achieved due to the use of the new rKDDR-plus antigen.

## Introduction

The leishmaniasis is a complex group of infectious parasitic diseases that presents a broad spectrum of clinical manifestations. The visceral form of leishmaniasis, or kala-azar, is considered one of the most lethal and neglected diseases in the world [1]. Visceral leishmaniasis (VL) is endemic in many countries representing a serious public health problem [2]. It is estimated a total of 0.2 to 0.4 million cases of VL in the world and 50,000 to 90,000 new cases each year [3,4]. In the Americas and Southern Europe, the VL presents zoonotic character, being the dog its main reservoir in urban areas. In these regions the VL is considered a disease of great human and veterinary medical importance [5]. In order to reduce the disease morbidity and lethality rates, the World Health Organization (WHO) recommends the joint use of several control strategies aimed at the main agents involved in this pathology [6]. One of the control strategies involves the early and accurate diagnosis for effective treatment [7].

Serological methods are important allies in the diagnostic of the VL, since this disease is characterized by a large production of specific antibodies against parasite antigens and by the stimulation of the polyclonal B cells in hosts [8–10]. Serological techniques, such as enzyme-linked immunosorbent assay (ELISA) and immunochromatographic test (ICT), are relatively simple, easy to execute, have low-cost and fast result, and can be performed manually or be automated [11,12]. The ICT still has the advantage of being technology and skilled labor independent, making this test an excellent tool for mass screening in the field [12]. These techniques allow the use of different types of antigens, such as the crude or soluble extract of *Leishmania* ssp. or recombinant proteins [13]. The sensitivity and specificity these techniques vary according to the antigen used [14]. Despite the numerous advantages, the serological tests have limitations, such as the detection of asymptomatic dogs and individuals or in early stage of the disease and the occurrence of cross-reactions with related diseases [15]. Recombinant molecules have emerged as an alternative to improve the quality of serologic diagnostics [16], with varying sensitivity and specificity [17–19].

Studies with protozoa show that antigens that have tandem repeat (TR) motifs in their sequence are possible targets for diagnosis, since they are usually highly antigenic B-cell epitopes [20–22]. Several TR proteins have also been reported as candidates for serodiagnosis of VL, as rK39 [23], A2 [24], rK26 [25], rKDDR [26], in addition to others identified by serological screening from an expression library of *L*. *infantum* [20].

One of the main antigens currently used in the diagnosis of VL is the rK39, composed of an immunogenic epitope that presents 6.5 copies of a tandemly arrayed 39 amino acid repeat, encoded by a kinesin-related gene from *L*. *infantum* [23]. The rK39 stands out from the other antigens described in the literature for its high capacity to discriminate patients with VL from healthy patients [16,23,27,28]. However, its sensitivity and specificity vary a lot according to the diagnostic method used and depending on the geographic region [29,30]. Recently, a new antigen named rKDDR, kinesin-derived from *L*. *infantum* and containing 8.5 TR motifs of the 39 amino acids, was evaluated for the diagnosis of VL. The rKDDR showed greater sensitivity and specificity when compared to rK39, both with human and dogs sera [26]. The promising relationship of these antigens in the VL diagnosis is probably associated with the presence of these TR regions, which seem to confer a greater antigenic capacity to proteins, being preferentially recognized by the antibodies present in the sera of patients [20,31].

The influence of the number of TR domains of a protein on antibody reactivity was previously reported by Goto et al. [32]. These authors showed that the antigenicity of these proteins is possibly influenced by their repetitive composition and by their upregulated expression in *Leishmania* amastigotes, which is the developmental stage in the mammalian host [32]. However, the effect of the number of these TR motifs on the diagnostic performance of an antigen remains unclear. Therefore, the objective of this study was to evaluate the impact of the progressive increase in the number of repetitive motifs in a recombinant protein for the diagnosis of VL. For this, we assessed the performance of a new kinesin-derived antigen from *L*. *infantum*, named rKDDR-plus, composed of an increased succession of TR sequences of 39 amino acids, totaling 15.3 repetitive blocks. Comparative serological tests were performed (ELISA and ICT) between the protein rKDDR-plus and the antigens also derived from kinesin, rKDDR and rK39, and the diagnostic efficiency was evaluated.

## Methods

### Ethics statement

To use of human sera in this study was submitted and approved by Human Research Ethics Committee (protocol number CAAE—00842112.2.0000.5149) of the Federal University of Minas Gerais. Participating individuals signed an Informed Consent Form agreeing to participate in the study. For patients younger than 18 years of age, the parents signed the Informed Consent Form, consenting to the child’s participation. Approval for use of the animal sera was obtained from the Ethics Committee on Animal Use (protocol number 44/2012) of the Federal University of Minas Gerais.

### Design and cloning of the KDDR-plus synthetic gene

The KDDR-plus synthetic gene was designed based on the nucleotide sequence of the *L*. *infantum* kinesin gene (LINF_140017300), which was obtained from the TriTrypDB database (http://tritrypdb.org). This gene was synthesized by the GenScript company (USA) in pUC57 cloning plasmid. The KDDR-plus synthetic gene includes the nucleotides 1,198 to 2,985 of *L*. *infantum* kinesin gene, totalizing 1,788 base pairs (bp). This sequence encodes 15.3 repeats of 39 amino acids presents in LINF_140017300 gene. Sites of restriction enzymes *Nhe*I and *Xho*I were added at 5’ and 3’ ends of the synthetic gene, respectively, to facilitate the subcloning of the gene into the pET28a-TEV expression vector (CeBiME, Campinas/SP, Brazil) and a stop codon (TAA) was added at 3’ end. The original codons of synthetic gene were optimized for the protein translation into *Escherichia coli* bacteria used as host cell for heterologous protein expression.

The pUC57/KDDR-plus plasmid was digested with the restriction enzymes to release the synthetic gene that was subsequently subcloning using the restriction sites of the pET28a-TEV plasmid. *E*. *coli* BL21 Star competent cells were transformed with the pET28a-TEV/KDDR-plus recombinant plasmid by electroporation. Correct gene insertion was confirmed by colony PCR and Sanger automated sequencing, performed by the Macrogen company (Seul, South Korea), using the sequencer ABI Prism 3730xl DNA Analyzer (Applied Biosystems, USA) and the T7 forward and T7 reverse primers to the pET28a-TEV vector.

### Heterologous expression and purification of the recombinant protein rKDDR-plus

The expression of rKDDR-plus in transformed *E*. *coli* cells was induced after the addition of 1 mM isopropyl-β-D-thiogalactopyranoside (IPTG) (Thermo Fisher Scientific, Brazil) and incubation for 3 h at 37°C at 180 rpm, using the shaker Maxq 400 (Thermo Scientific, USA). After centrifugation, the bacterial cells were resuspended in buffer A (20 mM NaH_2_PO_4_; 500 mM NaCl; 30 mM imidazole) in 10% of the initial volume of the culture. The cells were lysed with lysozyme (100 μg/mL) for 30 min followed of 5 cycles of mechanical lysis in the EmulsiFlex-C3 homogenizer (AVESTIN, Canada), using pressure peaks between 15,000 to 20,000 psi. The suspension was centrifuged at 6,000x*g* for 1 h at 4°C. Samples referring to the soluble and insoluble fraction were analyzed by sodium dodecyl sulfate polyacrylamide gel electrophoresis (SDS-PAGE) [33] for determined the solubility of the recombinant protein. Fractions of the cell extract before (0 h) and after (3 h) the addition of IPTG were also analyzed by SDS-PAGE.

The recombinant protein was purified by Ni^2+^ affinity chromatography, using the His-Trap HP column (GE Healthcare Life Science, Brazil) coupled to an ÄKTA Prime Plus system (GE Healthcare Life Science, Brazil). The rKDDR-plus protein was eluted with a linear gradient from 0 to 100% of buffer B (NaH_2_PO_4_ 20 mM; NaCl 500 mM; imidazole 500 mM) in a volume of 10 mL. To assess the purity and yield of the purified recombinant protein, the fractions obtained were analyzed by SDS-PAGE and the final concentration was indirectly determined by the BCA Protein Assay (Thermo Fisher Scientific, USA), according to manufacturer’s instructions.

### Bioinformatics analyses

The amino acid sequence of the rKDDR-plus protein was submitted to the BepiPred program (http://www.cbs.dtu.dk/services/BepiPred-1.0), with a cut-off of 1.0 (sensitivity < 0.25; specificity > 0.91), to prediction of linear epitopes of B-cells [34], and IUPred program (http://iupred.elte.hu/), with cut-off of 0.5 (score range of 0.0–1.0), to prediction of the protein structural disorder [35]. The theoretical molecular weight and isoelectric point (pI) were analyzed through the ProtParam program (https://web.expasy.org/protparam/).

### Serum samples

#### Human sera

Sera of 126 individuals were used in this study: 50 samples from patients with human VL (HVL) obtained from the University Hospital Clemente of Faria (Montes Claros, Minas Gerais State, Brazil), endemic region located in southeastern Brazil. The *L*. *infantum* infection was determined, from bone marrow samples, by the parasitological method (detection of the parasite) using Giemsa stain, and by specific qPCR assays for kDNA from the *Leishmania* parasite [36]. Samples from 54 patients chronically infected with *Trypanosoma cruzi* (Tc), to evaluate cross reactivity, were obtained from the University Hospital Clemente of Faria (Montes Claros, Minas Gerais State, Brazil) with infection confirmed by combination of positivity in Chagatest recombinant ELISA v.3.0 kit (Wiener Lab, Argentina) and the Chagatest Indirect Hemagglutination Assay (IHA) (Wiener Lab, Argentina). In addition, 22 sera from healthy individuals were used as negative control (NC) from an endemic area for VL and with negative results for *Leishmania* in tissue smears (bone marrow).

#### Canine sera

A total of 180 canine sera were used in the study. Sixty serum samples were collected in an endemic region for canine VL (CVL) (Montes Claros, Minas Gerais State, Brazil), and 36 samples from healthy dogs were used as non-endemic controls (NC), both confirmed by microscopic analysis of bone marrow aspirates. For cross-reactivity assessment of the tests were used 48 sera from dogs experimentally infected with *T*. *cruzi* (Tc), 27 dogs naturally infected with *Babesia* sp. (Bab) and 9 with *Ehrlichia* sp. (Ehr). Serum samples from *T*. *cruzi* were obtained from Department of Clinical Analysis of the School of Pharmacy/UFOP. Samples from dogs naturally infected with *Babesia* sp. and *Ehrlichia* sp. were obtained from a private veterinary laboratory (Contagem/Minas Gerais State, Brazil) and positivity to both the parasites was confirmed by parasitological techniques (blood smears).

### Crude soluble antigen of *L*. *infantum*

The *L*. *infantum* reference strain MHOM/BR/1974/PP75 was used in this study. *L*. *infantum* promastigotes were cultured at 24°C in Schneider’s medium (Sigma-Aldrich, USA), supplemented with 10% of inactivated bovine fetal sera, containing 100 U/mL de penicillin and 100 μg/mL de streptomycin (Gibco/Thermo Fisher Scientific, USA). Approximately 5x10^8^ parasites were washed and recovered in 1 mL de PBS followed of 15 lysis cycles of freezing in liquid nitrogen and thawing at 37°C.

### Enzyme-linked immunosorbent assay (ELISA)

The performance of the rKDDR-plus, rKDDR [26], rK39 [23] and rK26 [25] proteins and crude soluble antigen (CSA) of *L*. *infantum* was evaluated by ELISA. The rKDDR protein was obtained and purified as described by Dhom-Lemos et al. [26]. The rK39 and rK26 antigens were kindly provided by Steven G. Reed (Infectious Disease Research Institute—IDRI, Seattle, Washington). The optimal coating concentrations of the recombinant antigens used in this work were determined empirically through a titration optimization using these proteins. The concentration of CSA was performed as previously described [23]. All antigens were diluted in 100 μL carbonate buffer [Na_2_CO_3_ 15 mM (Synth, Brazil); NaHCO_3_ 34 mM (Merck, Brazil) pH 9.6] in amounts of 50 ng per well to the rKDDR-plus and rKDDR proteins, and 100 ng per well to the rK39 and rK26 proteins and CSA. ELISA microplates of 96 wells (Costar, USA) were coated with the antigens and incubated for 16 h at 4°C. To avoid non-specific links, the plate was blocked with 2% BSA (Fitzgerald, USA) in PBS pH 7.2 for a period of 2 h at room temperature. Human and canine serum samples diluted 1:100 in PBS containing 0.05% Tween 20 (PBS-T) were added (100 μL/well) and incubated for 16 h at 4°C. Plates were washed in wash buffer (PBS-T) five times and incubated with 100 μL of the anti-human IgG (Fc specific) − peroxidase antibody produced in goat (#A0170, Sigma-Aldrich, USA) or anti-dog IgG (whole molecule)–peroxidase antibody produced in rabbit (#A6792, Sigma-Aldrich, USA), diluted in PBS-T at 1:5,000 to human sera or 1:2,500 to canine sera, and incubated at 37°C for 1 h and 30 min. After a final wash step of the plates, the enzyme reaction was developed with 100 μL/well of substrate solution [0.1 M citric acid, 0.2 M Na_2_PO_4_, 0.05% *o*-phenylenediamine dihydrochloride (OPD) (Sigma- Aldrich, USA) and 0.1% H_2_O_2_] for 10 min at 37°C. The reaction was stopped using 50 μL of 4N sulfuric acid (H_2_SO_4_) and the plates were read at 492 nm in an automated microplate ELISA reader (Versamax, Molecular Devices, USA).

### Rapid immunochromatographic dipstick test (ICT)

#### Development of the ICT/KDDR-plus

An ICT prototype was developed in partnership with Safetest Diagnósticos (Brazil) based on the rKDDR-plus protein (S1A Fig). The device is formed by an initial region containing a sample application pad, which adsorbs the sample and distributes it homogeneously to the membrane, followed by a conjugate release membrane, in which there are colloidal gold nanoparticles, colored in red, linked to the rKDDR-plus antigen or the mouse IgG antibody. Soon after, the rKDDR-plus antigen is immobilized on the membrane within the test area, followed by the control area, in which there is a capture reagent (anti-mouse IgG), which acts as a validation of the test reactivity. After the control line, there is an absorbent pad that decreases the background color by increasing the sample flow volume (S1A Fig). The device is stored in the dark in aluminium bags containing silica at room temperature until use.

#### ICT/rKDDR-plus for HVL diagnosis

Assays to detect anti-leishmanial antibodies were carried with ICT/KDDR-plus prototype, using 69 human sera randomly selected from the serological panel previously described, being 29 serum samples from patients infected with *Leishmania* (HVL); 20 from patients chronically infected with *T*. *cruzi* (Tc); and 20 from healthy individuals (NC). The performance of the ICT/rKDDR-plus (Safetest Diagnósticos, Brazil) was evaluated and compared to IT LEISH ICT by DiaMed AG (Cressier, Switzerland) and distributed by DiaMed Latin America S.A. (Lagoa Santa, Brazil), based on the rK39 antigen, following the manufacturer instructions. The evaluation of the test was performed in duplicate and the qualitative analysis of the results was performed by the naked eye after 20 min of the beginning of the test by two independent evaluators. The results were established by presence (positive result) or absence (negative result) of a red line in the test area, under the condition that a red line could be visualized in the control area (S1B Fig).

### Data analysis

The ELISA data (S1 File) were analyzed using the Software GraphPad Prism 5.0 (GraphPad Prism Inc., USA) and MedCalc with confidence intervals of 95%. Through the receiver operating characteristic (ROC) curve was possible to evaluate all the combinations of the sensibility and specificity and to determine a cut-off value for each antigen tested. For the ROC curve analysis, the infected group was composed of VL samples and the control group was composed of non-VL samples. Area under the curve (AUC) was calculated and diagnostic performance was established. The positive predictive values (PPV) and negative predictive values (NPV) as well as the accuracy (AC) of the tests were calculated by the ratio of true-positive (TP) and false-negative (FN). The degree of agreement between the serological tests (ELISA and ICT) and the parasitological test (biopsy, aspirate or PCR) was estimated by kappa index (k) with 95% confidence interval and classified according to the Fleiss scale: 0.00–0.20, poor; 0.21–0.40, fair; 0.41–0.60, moderate; 0.61–0.80, good; 0.81–0.99, very good; and 1.00, perfect.

## Results

### Composition and molecular analysis of the rKDDR-plus

The complete nucleotide sequence of the *L*. *infantum* kinesin gene (LINF_140017300) has 10,830 bp and encodes a protein with 3,609 amino acids, divided into two different portions: a non-repetitive amino acid sequence at the beginning of the protein and a repetitive region containing a tandem block of 39 amino acids, with some degenerations in the sequence. The KDDR-plus synthetic gene was designed based on the sequence encoding 15.3 tandem repeats of 39 amino acids present in the kinesin protein, totalizing 1,788 nucleotides.

**Fig 1:**
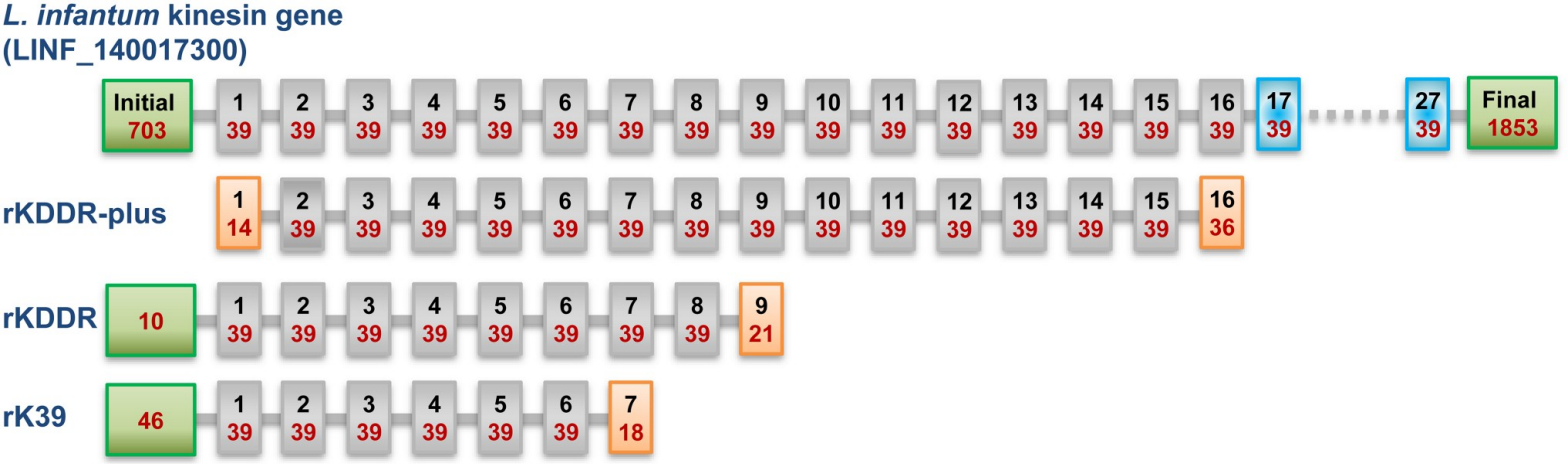
Comparison of the structure of the rKDDR-plus and other antigens based on the kinesin protein of *L*. *infantum*. The kinesin-derived proteins used in the comparison were rKDDR-plus, rKDDR [26], and rK39 [23]. The red numbers represent the amount of amino acids present in each block and the black numbers indicate the position of the block. The green blocks represent the non-repetitive portion of amino acids present in the kinesin proteins. The gray blocks represent the repetitive motif units of 39 amino acids present in each protein. The blue blocks indicate repetitive blocks present in the kinesin protein, but which have variations of certain amino acids in relation to the consensus sequence of 39 amino acids described by Burns et al. [23]. The orange blocks represent the incomplete repetitive motif units of amino acids.

The rKDDR-plus protein is formed only by the repetitive portion of *L*. *infantum* kinesin protein, unlike other proteins derived from kinesin and widely used in the diagnosis of VL in the world, such as rKDDR [26] and rK39 [23] (Fig 1). In addition, this antigen has a higher number of motif repetitions of 39 amino acids (15.3 repeats), when compared with the other proteins derived from kinesin: rK39 (6.5 repeats) and rKDDR (8.5 repeats).

The synthetic KDDR-plus gene had optimized *in silico* codons, and then cloned into the pET28a-TEV expression vector, which contains a sequence encoding a histidine tag. After cloning, a single ORF (Open Reading Frame) with 1,848 bp was obtained, which encodes 615 amino acids (S2 Fig), with a theoretical molecular mass of 68 kDa and a pI of 4.56. Thus, 97% (596 amino acids) of the protein sequence consists exclusively of repetitive motifs, while the remaining 3% (19 amino acids) originate from the plasmid sequence.

The construct pET28a-TEV/KDDR-plus was transformed into *E*. *coli* BL21 Star and the recombinant rKDDR-plus protein was expressed by IPTG induction and presented a high yield after 3 h of induction (S3A Fig). The solubility test indicated that the protein is soluble and can be purified under native conditions (S3A Fig). After purification by affinity chromatography, the rKDDR-plus protein was obtained with high level of purity. Analysis of the purified fraction revealed the presence of a highly expressed 68 kDa band, referring to rKDDR-plus (S3B Fig), with estimated yield of approximately 3 mg of protein per 1 L bacterial culture.

### Prediction of linear B-cell epitopes and structural disorder

To evaluate the antigenic potential of rKDDR-plus, we predicted the linear B-cell epitopes present in the protein using the BepiPred 1.0 tool. In addition, the degree of structural disorder along the rKDDR-plus protein was determined using the IUPred tool in order to verify regions with no secondary structures.

The data obtained in predictions indicate the presence of a large number of linear B-cell epitopes in rKDDR-plus (S4 Fig), suggesting that this protein is probably capable of interacting with lymphocytes of the host immune system and can be applied as a possible target for the VL diagnosis. The presence of a large region of structural disorder was also observed along the length of the rKDDR-plus protein sequence (S4 Fig). These results suggest that the B-cell epitopes present in the protein are available to be recognized by the host immune system, since they are not involved in internal secondary interactions within the protein.

### ELISA tests with rKDDR-plus for detection of IgG antibodies in patients with human visceral leishmaniasis

**Fig 2:**
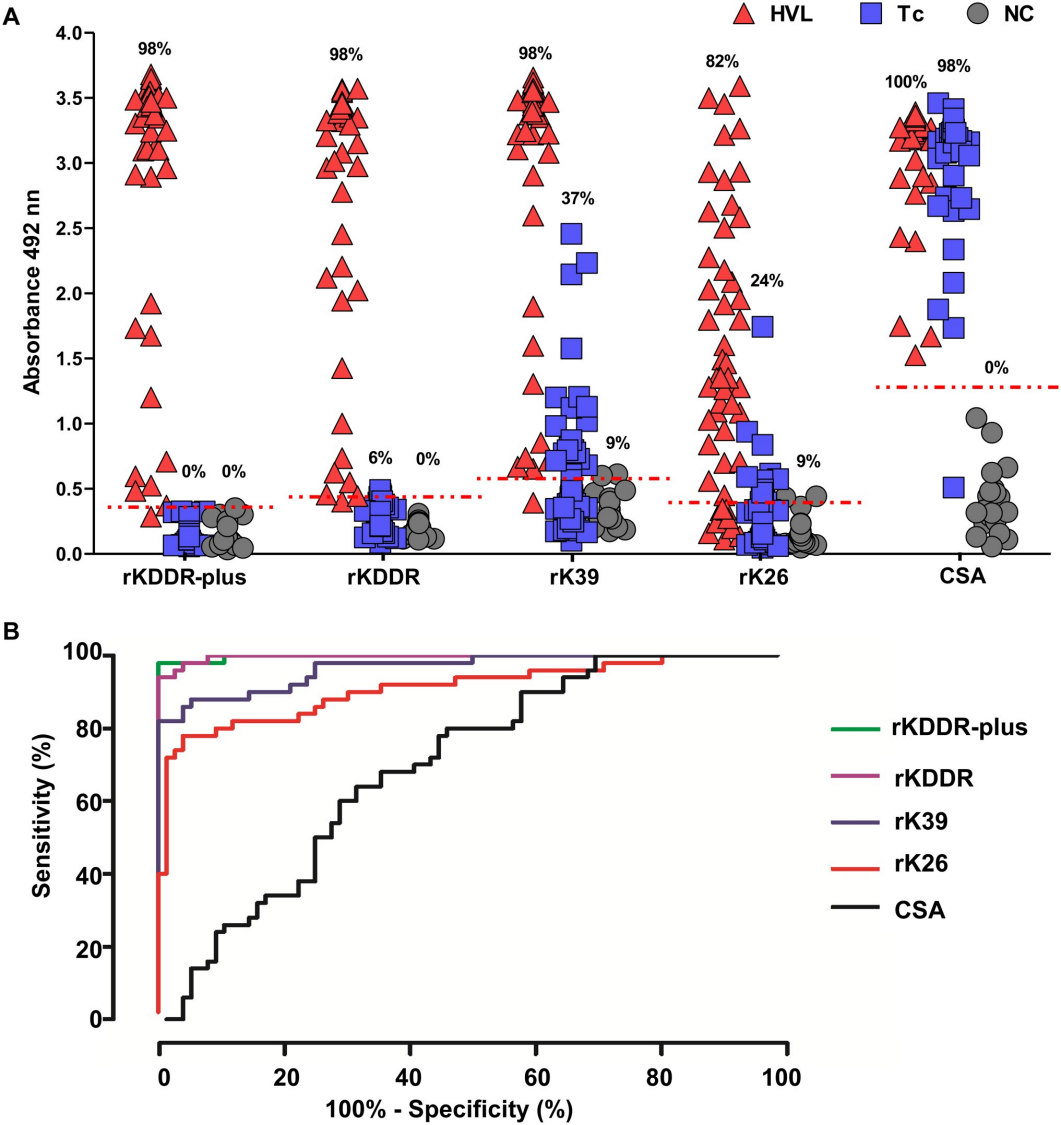
Evaluation of the recombinant antigens and *L*. *infantum* crude extract for the serodiagnosis of human visceral leishmaniasis (HVL). **(A)** Comparison of the performance of ELISA with the rKDDR-plus, rKDDR, rK39 and rK26 antigens and the crude soluble antigen (CSA) of *L*. *infantum* was performed using serum samples from the following groups: patients infected with *Leishmania* (HVL, n = 50); healthy individuals used as negative control (NC, n = 22); and patients chronically infected with *T*. *cruzi* (Tc, n = 54), to evaluate cross reactivity. The dashed red line represents the cut-off determined by the ROC curve of each test. The index above each column in the plot indicates the percentage of samples that are above the cut-off. **(B)** Analysis of ROC curve of each human serological test obtained for ELISA using rKDDR-plus, rKDDR, rK39 and rK26 antigens and the CSA. The sensitivity, specificity and area under the curve (AUC) were determined by ROC curve.

The recognition potential of the rKDDR-plus antigen for the HVL diagnosis was determinate by assessing the reactivity of this protein in ELISA followed by the comparison of its performance with the antigens also derived from kinesin, rKDDR [26] and rK39 [23], and also with the rK26 protein [25] and the CSA, used as controls (Fig 2A). The rK26 antigen is derived from the hydrophilic surface protein B, which contains 11 repeats of 14 amino acids that comprise 64% of the total protein [25]. This protein was used as a control, as it is a repetitive protein not derived from kinesin.

The rKDDR-plus, rKDDR and rK39 antigens showed the same percentage of serum recognition from patients with HVL (98%) (Fig 2A). In turn, rK26 and CSA showed 82% and 100% recognition of the positive sera, respectively. However, rKDDR-plus was the only antigen that did not cross-reaction with sera from chagasic patients compared to the other antigens evaluated. In addition, the rK39 and rK26 antigens also identified sera from control group individuals (not infected) as positive. The cut-off of ELISA plots was determined for each antigen from the ROC curve (Fig 2B). The cut-off for the rKDDR-plus, rKDDR, rK39 and rK26 antigens and the CSA was 0.3611, 0.4437, 0.5839, 0.3977 and 1.284 absorbance units (AU), respectively.

Analysis of the AUC values using ROC curves confirmed the better performance of the rKDDR-plus antigen (AUC = 0.9979), suggesting that this protein presents a great potential to discriminate the cases of patients with and without the HVL (Table 1). According to the Fleiss scale, the degree of agreement of rKDDR-plus (k = 0.983), with the parasitological/qPCR reference test, was classified as “very good”, suggesting to be a diagnostic test of excellent quality (Table 1). The sensitivity, specificity, positive and negative predictive value, as well as accuracy was determined for the five antigens tested (Table 1). The rKDDR-plus, rKDDR and rK39 antigens had the same sensitivity (98%), while rK26 and the CSA showed 82% and 100%, respectively. However, the rKDDR-plus ELISA showed the best results of specificity (100%), PPV (100%), NPV (98.70%) and accuracy (99.21%), when compared to other antigens that presented lower values (Table 1).

**Table 1:** Diagnostic performance of ELISA tests using human serum samples from patients with visceral leishmaniasis (VL) and non-VL individuals.

Antigen	PPV (%)	NPV (%)	Sen (%) [CI 95%]	Spe (%) [CI 95%]	AC (%)	AUC	Kappa [CI 95%]	Agreement
**rKDDR-plus**	100.00	98.70	98.00 [89.35–99.95]	100.00 [95.26–100.0]	99.21	0.9979	0.983 [95.1–100.0]	Very good
**rKDDR**	94.23	98.65	98.00 [89.35–99.95]	96.05 [88.89–99.18]	96.83	0.9971	0.934 [87.1–99.8]	Very good
**rK39**	69.01	98.18	98.00 [89.35–99.95]	71.05 [59.51–80.89]	81.75	0.9655	0.644 [52.0–76.9]	Good
**rK26**	73.21	87.14	82.00 [68.56–91.42]	80.26 [69.54–88.51]	80.95	0.9100	0.610 [47.1–75.0]	Good
**CSA**	48.00	92.31	100.0 [92.89–100.0]	30.26 [20.25–41.87]	57.14	0.6976	0.236 [12.5–34.6]	Fair

PPV: positive predictive value; NPV: negative predictive value; Sen: sensitivity; Spe: specificity; AC: accuracy; AUC: area under curve; CI: confidence interval; CSA: crude soluble antigen. Reference test: parasitological/qPCR.

### Serological recognition of rKDDR-plus by ELISA for the diagnosis of canine visceral leishmaniasis

**Fig 3:**
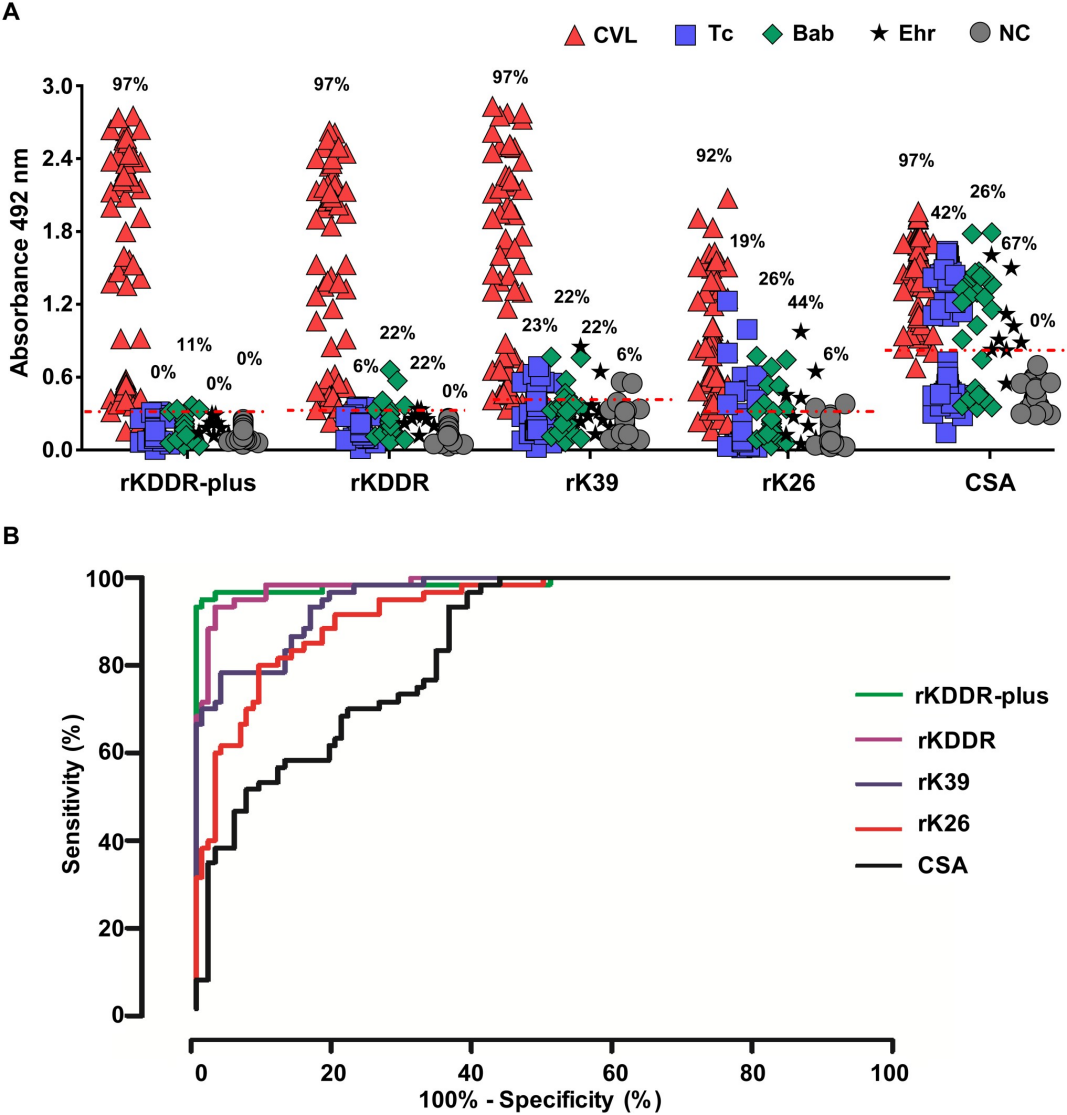
Evaluation of the recombinant antigens and *L*. *infantum* crude extract for the serodiagnosis of canine visceral leishmaniasis (CVL). **(A)** Comparison of the performance of ELISA with the rKDDR-plus, rKDDR, rK39 and rK26 antigens and the crude soluble antigen (CSA) was performed using canine sera from the following groups: dogs infected with *Leishmania* (CVL, n = 60); non-infected healthy dogs (NC, n = 36); dogs naturally infected with *Babesia* sp. (Bab, n = 27) and *Ehrlichia* sp. (Ehr, n = 9); and dogs experimentally infected with *T*. *cruzi* (Tc, n = 48), for cross-reactivity assessment. The dashed red line represents the cut-off determined by the ROC curve of each test. The index above each column in the plot indicates the percentage of samples that are above the cut-off. **(B)** Analysis of ROC curve of each canine serological test obtained for ELISA using rKDDR-plus, rKDDR, rK39 and rK26 antigens and the CSA. The sensitivity, specificity and area under the curve (AUC) were determined by ROC curve.

All the antigens tested by ELISA had the same percentage of serological recognition (97%) of sera from dogs with CVL, except rK26 (92%) (Fig 3A). However, rKDDR-plus presented a lower percentage of cross-reaction between the tested antigens, only 11% with babesiosis. For other sera used as control, this antigen showed no cross-reactivity. The other antigens showed a high percentage of cross-reactivity with babesiosis, Chagas disease and ehrlichiosis. The rK39 and rK26 antigens also showed a reaction with sera from healthy dogs (negative control). The ROC curve (Fig 3B) was used to determine the cut-off of each antigen, obtaining the following values in absorbance units (AU): 0.3147 (rKDDR-plus), 0.3248 (rKDDR), 0.4087 (rK39), 0.3251 (rK26) and 0.8314 (CSA).

The rKDDR-plus showed the highest AUC value (0.9889), followed by rKDDR, rK39, rK26 and the CSA, which presented the values 0.9871, 0.9631, 0.9358 and 0.8608, respectively (Table 2). All recombinant antigens showed the same sensitivity (96.67%), except rK26 (91.67%). However, rKDDR-plus has a higher specificity (97.50%) when compared to the rKDDR (90.83%), rK39 (82.50%), rK26 (81.67%) antigens and the CSA (64.17%) (Table 2). The rKDDR-plus also obtained higher PPV (95.08%), NPV (98.32%) and AC (97.22%) compared to the other antigens (Table 2). The highest kappa concordance index was obtained again by rKDDR-plus (0.938), showing a “very good” degree of agreement, suggesting its potential use for canine serodiagnosis.

**Table 2:** Diagnostic performance of ELISA tests to detect antibodies against canine visceral leishmaniasis (CVL).

Antigen	PPV (%)	NPV (%)	Sen (%) [CI 95%]	Spe (%) [CI 95%]	AC (%)	AUC	Kappa [CI 95%]	Agreement
**rKDDR-plus**	95.08	98.32	96.67 [88.4–99.59]	97.50 [92.87–99.48]	97.22	0.9889	0.938 [88.4–99.2]	Very good
**rKDDR**	84.06	98.20	96.67 [88.4–99.59]	90.83 [84.19–95.33]	92.78	0.9871	0.843 [76.2–92.5]	Very good
**rK39**	73.42	98.02	96.67 [88.4–99.59]	82.50 [74.50–88.83]	87.22	0.9631	0.734 [63.4–83.3]	Good
**rK26**	71.43	95.15	91.67 [81.6–97.24]	81.67 [73.57–88.14]	85.00	0.9358	0.685 [57.8–79.2]	Good
**CSA**	57.43	97.47	96.67 [88.4–99.59]	64.17 [54.90–72.71]	75.00	0.8608	0.520 [41.0–62.9]	Moderate

PPV: positive predictive value; NPV: negative predictive value; Sen: sensitivity; Spe: specificity; AC: accuracy; AUC: area under curve; CI: confidence interval; CSA: crude soluble antigen. Reference test: parasitological.

### Evaluation of ICT/rKDDR-plus for the diagnosis of human visceral leishmaniasis

**Fig 4:**
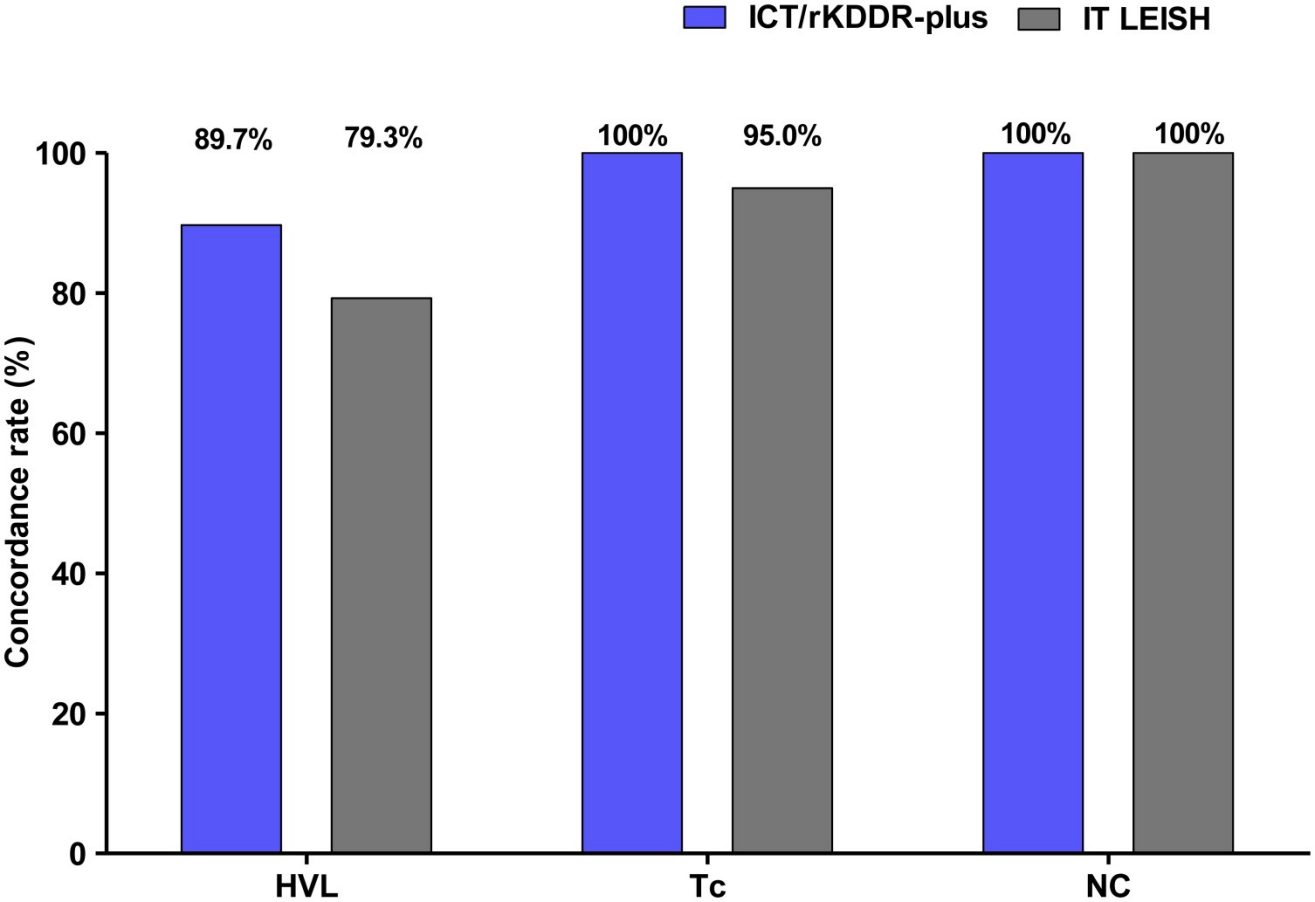
Concordance rate of the rapid immunochromatographic test results with reference standard for diagnosis of human visceral leishmaniasis (HVL) with the serum samples. The comparison of the performance between the rapid tests ICT/rKDDR-plus (Safetest Diagnósticos) and IT LEISH (DiaMed AG) was performed using 69 human serum samples from patients infected with *Leishmania* (HVL, n = 29); patients chronically infected with *T*. *cruzi* (Tc, n = 20), to evaluate cross reactivity; and healthy individuals used as negative control (NC, n = 20). Reference test: parasitological/qPCR.

The ICT based on the rKDDR-plus recombinant antigen (Safetest Diagnósticos, Brazil) had its diagnostic performance evaluated and compared to the IT LEISH ICT, produced by DiaMed AG (Cressier, Switzerland). Both diagnostic tests were assessed with 69 human serum samples (Fig 4 and Table 3). The ICT/rKDDR-plus detected 26 of 29 sera tested from patients infected with *L*. *infantum*, resulting in a sensitivity of 89.7%. No false positive result was attributed to ICT/rKDDR-plus, resulting in a specificity of 100%. The accuracy, PPV and NPV of ICT/KDDR-plus were of 95.7%, 100.0% and 93.0%, respectively. In comparison, IT LEISH test identified only 23 of the 29 sera tested from patients with HVL, representing a sensitivity of 79.3%. All individuals of the control group (uninfected) were correctly diagnosed, but when sera from patients with Chagas disease were analyzed, the IT LEISH rapid test identified 1 of 20 sera tested (Fig 4). These results represent a specificity of 97.5%, with accuracy, PPV and NPV of 89.9%, 95.8% and 86.7%, respectively (Table 3). The accuracy obtained by IT LEISH against the kappa agreement criterion was considered “good” at kappa index of 0.787, whereas ICT/rKDDR-plus was considered “very good”, obtaining a kappa index of 0.909 (Table 3).

**Table 3:** Diagnostic performance of immunochromatographic tests with human sera from patients infected with *Leishmania infantum*, *Trypanosoma cruzi* and healthy controls.

Test	Positive samples	Sen (%)	NPV (%)	Negative samples	Spe (%)	PPV (%)	AC (%)	Kappa [CI 95%]	Agreement
**ICT/rKDDR-plus**	26/29	89.7	93.0	40/40	100.0	100.0	95.7	0.909 [81.0–100]	Very good
**IT LEISH**	23/29	79.3	86.7	39/40	97.5	95.8	89.9	0.787 [63.9–93.5]	Good

PPV: positive predictive value; NPV: negative predictive value; Sen: sensitivity; Spe: specificity; AC: accuracy; CI: confidence interval. Reference test: parasitological/qPCR.

## Discussion

In this study, we evaluated the performance of a new recombinant antigen rKDDR-plus, composed of an increased number of a repetitive motif of 39 amino acids, presents in the *L*. *infantum* kinesin protein, totaling 15.3 copies. The performance of this new antigen was compared with two other kinesin-derived recombinant antigens described in the literature, rK39 [23] and rKDDR [26], which have a smaller number of repetitive motifs (6.5 and 8.5 copies, respectively), in addition to being also composed of a non-repetitive region of the kinesin protein. In contrast, the rKDDR-plus protein has only the repetitive region of the kinesin protein. The use of TR proteins in the diagnosis of VL was studied by Goto et al. [20]. In another study, Goto et al. [32] performed ELISAs using *Leishmania* recombinant TR proteins and demonstrated that the copy number of the repeat affects the binding affinity between antigens and antibodies, due to thermodynamic binding kinetics. However, the influence of the number of repetitions of a sequence on the diagnostic performance has not yet been evaluated.

One of the current challenges for the VL control revolves around improving the performance of diagnostic tests for human and veterinary use. Several studies have focused on the development of more accurate serodiagnosis, through replacing CSA by recombinant proteins [16,37–41]. Until the early 1990s, the targets used in the serological tests were the crude or soluble antigens of promastigotes or amastigotes forms of *Leishmania* ssp., which has a high sensitivity for the detection of specific antibodies in human and canine sera, but with a very limited specificity [42,43]. Fraga et al. [44] showed that the CSA in the ELISA test showed a sensitivity of 97%, but a very limited specificity, 26%. Likewise, another study also showed a good sensitivity (100%) when using CSA, but a low specificity (68%) [45]. Corroborating these results, we also found a low specificity for both human sera (30.26%) and canine sera (64.17%) in ELISA using CSA.

Recombinant antigens appear as an alternative to the use of CSA, because they are specific molecules of the parasite that have high affinity with antibodies present in the vast majority of biological samples and, unlike the CSA, have an excellent specificity [23,39] In addition to improving the specificity of the tests, without compromising their sensitivity, recombinant proteins allow large-scale and standardized production, independent of parasite cultures, with high efficiency and remarkable purity at relatively low costs [37,38]. Several recombinant molecules have been characterized and evaluated for use with samples from humans and dogs in the VL diagnosis. Among them, it is worth mentioning the proteins with TR motifs in their sequence, as the kinesin-derived antigens of *L*. *infantum* (rK39 [23] and rKDDR [26]) and the hydrophilic acylated surface protein (rK26) [25].

Regions with successive copies of the same amino acid sequence have been reported in several eukaryotic proteomes, including protozoa such as *Plasmodium* spp., *Trypanosoma* spp. and *Leishmania* spp. [20,22,46,47]. Some studies have shown that individuals with VL or other infections, such as trypanosomiasis and malaria, have high production of specific antibodies against these repetitive regions [48–51]. Goto et al. [20] showed that TR proteins are more potent as immunodominant B-cell antigens, being more highly recognized by VL patient sera than non-TR proteins. Another study shows that among the reasons for TR proteins to be more antigenic may be related to the exposure of several identical sequences with a single epitope to the host’s immune system, generating an immunodominance [20,52]. After evaluating the reactivity of TR proteins with plasma samples from patients with VL, Goto et al. [32] also demonstrated that the antibody binding to the antigen is stronger as the copy number of the repeats increases.

This is probably the reason why kinesin-derived proteins are recognized as good antigens for the VL diagnosis, since they have a highly immunogenic TR region composed of 39 amino acids, as shown in rK39 [23] and rKDDR [26]. We also analyzed *in silico* the presence of potential B-cell epitopes in the new antigen described here, rKDDR-plus. Throughout its sequence, we observed the presence of a large number of predicted linear B-cell epitopes coinciding with a region of structural disorder. These results suggest that the rKDDR-plus antigen is probably capable of interacting with lymphocytes of the host’s immune system. These epitopes can be more easily identified during antibody serological screening compared to molecules with fewer repetitions, thus compensating for the low prevalence of antibodies observed in some patients [53].

To evaluate the influence of the number of 39 amino acid motif repetitions from kinesin-derived proteins, we performed comparative ELISA assays with the three proteins. Although the sensitivity of the rK39, rKDDR and rKDDR-plus antigens were the same (98.00% for humans and 96.67% for dogs) in the ELISA, the specificity was proportional to the number of repetitive motifs present in the proteins. Considering that rK39 has 6.5 repeats, rKDDR 8.5 repeats, and rKDDR-plus 15.3 repeats, their specificities were 71.05%, 96.05% and 100.00% for human sera, and 82.50%, 90.83% and 97.50% for canine sera, respectively. Consequently, the antigen with the highest number of repetitive motifs (rKDDR-plus) was also the one that obtained the best accuracy results, with both sera, followed by rKDDR and rK39. Therefore, these results suggest that the increased presence of TR motifs can increase the diagnostic performance of serological tests for VL. The considerable cross-reactivity of the antigens tested with the sera from dogs infected with *Babesia* sp. and *Ehrlichia* sp. could be explained by a possible exposure of these animals to *Leishmania*, since the reference diagnosis used was the parasitological, which may have a lower sensitivity than serological. Even considering this possibility, rKDDR-plus showed an excellent result since this antigen did not show any cross-reactivity with *Ehrlichia* sp. and showed minor reactivity (only 11%) with *Babesia* sp., the lowest value among the tested antigens. In the case of dogs infected with *T*. *cruzi*, this possibility does not apply because the infection was made experimentally, with no possibility of contamination with *L*. *infantum*.

In the ELISA experiments, in addition to the CSA, the rK26 protein was also used as a control. This antigen has 11 repeats of a 14 amino acid sequence that can also be described as two alternating repeats of 7 amino acids (PKEDGH/RT and QKNDGDG) [25]. The rK26 has a sensitivity ranging from 21.3% to 96.8% and a specificity from 80% to 100% described in other studies [54–57]. Although the rK26 antigen also has an expressive number of repetitive motifs (11 repeats), greater than the number of repetitions of rK39 and rKDDR, it showed lower values of sensitivity and specificity for human sera (82.00% and 80.26%, respectively) and for canine sera (91.67% and 81.67% respectively), when compared to all antigens derived from kinesin—except for the specificity of rK39 that was lower with human sera. However, the 14 amino acid repetitive region of rK26 shares no homology with the 39 amino acid repetitive region of proteins derived from kinesin. Therefore, these results suggest that the diagnostic performance of a test depends on both the composition of the TR sequence and its number of copies.

Another advantage attributed to recombinant proteins is their adsorption capacity on various surfaces that can be used in the field. In the last decade, ICT has been used as diagnosis of VL in several countries [58,59]. This test combines the easy execution and rapid interpretation of the results, as well as the independence of laboratory infrastructure and specialized professionals for the execution [12]. Therefore, in this study, we developed and evaluated an ICT based on the rKDDR-plus protein, to be used in the rapid diagnosis of HVL. The sensitivity and specificity of the ICT/rKDDR-plus were 89.7% and 100.0%, respectively. The performance of ICT/rKDDR-plus was compared with the commercially available ICT IT LEISH, which is based on the rK39 antigen. In our analyses, ICT IT LEISH obtained a sensitivity of 79.3% and specificity of 97.5%. These results show that ICT/rKDDR-plus has a greater ability to detect the disease when it is present and to discard it when it is absent compared to commercial test. The sensitivity of ICT IT LEISH can vary from 81.1% to 96.2%, whereas its specificity varies between 96.6% to 98.7% [60–63]. Therefore, it is possible to conclude that the rapid test ICT/rKDDR-plus has great potential to be used as a diagnosis for HVL in field screening, since it presented a better diagnostic performance.

However, the results presented in this study on the use of rKDDR-plus in the VL diagnosis refer to an initial and relatively small-scale trial. In addition, the results obtained for NPV and PPV in the ELISA and ICT tests may differ depending on the prevalence of VL in the endemic areas being tested or sample set with different VL positivity rates. Therefore, tests with a greater number of samples, carried out in field, are in progress, and will allow us to verify points that could be improved in these diagnostic tests. Currently, the KDDR-plus antigen has been licensed by a company for the commercialization of ELISA and ICT for the diagnosis of human VL, and is in the process of transferring technology for veterinary use.

Therefore, the results obtained in this study show that the recombinant antigen rKDDR-plus is a highly promising candidate for VL diagnosis in both serological tests (ELISA and ICT), presenting the highest accuracy and specificity compared with others kinesin-derived antigens, which are currently used in the diagnosis of this disease. The best diagnostic performance of rKDDR-plus in serological tests is probably due to the increased presence of repetitive motifs in the protein.

## Supporting information

S1 Fig Rapid immunochromatographic lateral flow test (ICT) prototype using the rKDDR-plus antigen. **(A)** Schematic representation of the diagnostic ICT device. The three schematic strips represent the test steps: 1) sample application; 2) sample migration; 3) test result. **(B)** Photograph of representative results of ICT/rKDDR-plus strips tested with different human serum samples. HVL: test with sera from two patients infected with *Leishmania*; Tc: test with sera from two patients infected with *T*. *cruzi*; NC: test with two negative sera from healthy individuals. With negative serum samples for leishmaniasis (NC and Tc), only the control line is present, while with serum samples from VL patients, both control and test lines turned red.
(TIF)

S2 Fig Schematic representation of the DNA sequence and predicted amino acid sequence of the rKDDR-plus protein. Lowercase letters represent the nucleotide sequence of KDDR-plus and uppercase letters represent the translated protein sequence. The nucleotides and amino acids indicated in red at the ends of the sequences represent the portion derived from the plasmid pET28a-TEV, used in the expression of the protein in bacteria. The underlined nucleotides in purple correspond to the initiation codon; the underlined in yellow represent the histidine tag added to the protein to facilitate the purification process; the underlined nucleotides in blue correspond to the sites for the restriction enzymes; and the underlined in green represent the cleavage site for the TEV protease. The remainder of the underlined sequence shows the 15.3 repetitive motifs of 39 amino acids derived from the *L*. *infantum* kinesin protein, except for the first motif with 14 amino acids and the 16th motif with 36 amino acids. The ordinal numbers indicated on the left represent the number of repetitive motifs of rKDDR-plus. The numbers on the right represent the position of the nucleotides.
(TIF)

S3 Fig Analysis in polyacrylamide gel electrophoresis (SDS-PAGE) of the expression in bacteria and purification of rKDDR-plus protein. **(A)** Extracts of *E*. *coli* bacteria, BL21 Star strain, containing the plasmid pET28a-TEV/KDDR-plus, before (0h) and after (3h) induction of the recombinant protein with IPTG (1 mM). The bacterial extract was lysed and separated into soluble and insoluble fraction by centrifugation. The red arrow indicates the rKDDR-plus protein band after expression and solubility test. **(B)** After purification by affinity chromatography of the soluble fraction of the bacterial lysate, the purified fraction presented a band of approximately 68 kDa, corresponding to rKDDR-plus. MM: molecular mass marker; kDa: kilodalton.
(TIF)

S4 Fig Predictions of linear B-cell epitopes and structural disorder regions of rKDDR-plus protein. The dashed arrow corresponds to the complete amino acid sequence of the protein. The orange boxes correspond to the linear B-cell epitopes predicted by the BepiPred program, while the gray box corresponds to the prediction of structural protein disorder using the IUPred program. The value below each box corresponds to the score of each prediction.
(TIF)

S1 File ELISA data obtained in this study. Data used to make ELISA figures and tables.
(PDF)

S2 File STARD checklist. Reports of studies of diagnostic accuracy.
(DOCX)

## References

[1] Harhay MO, Olliaro PL, Costa DL, Costa CH. Urban parasitology: visceral leishmaniasis in Brazil. Trends Parasitol. 2011;27(9):403–9. doi: 10.1016/j.pt.2011.04.001 21596622

[2] Costa DN, Codeço CT, Silva MA, Werneck GL. Culling dogs in scenarios of imperfect control: realistic impact on the prevalence of canine visceral leishmaniasis. PLoS Negl Trop Dis. 2013;7(8):e2355. doi: 10.1371/journal.pntd.0002355 23951375 PMC3738479

[3] Alvar J, Vélez ID, Bern C, Herrero M, Desjeux P, Cano J, et al. Leishmaniasis worldwide and global estimates of its incidence. PLoS One. 2012;7(5):e35671. doi: 10.1371/journal.pone.0035671 22693548 PMC3365071

[4] Bi K, Chen Y, Zhao S, Kuang Y, John Wu CH. Current Visceral Leishmaniasis Research: A Research Review to Inspire Future Study. Biomed Res Int. 2018;2018:9872095. doi: 10.1155/2018/9872095 30105272 PMC6076917

[5] Shimozako HJ, Wu J, Massad E. The Preventive Control of Zoonotic Visceral Leishmaniasis: Efficacy and Economic Evaluation. Comput Math Methods Med. 2017;2017:4797051. doi: 10.1155/2017/4797051 28588642 PMC5447317

[6] Dinesh DS, Das ML, Picado A, Roy L, Rijal S, Singh SP, et al. Insecticide susceptibility of Phlebotomus argentipes in visceral leishmaniasis endemic districts in India and Nepal. PLoS Negl Trop Dis. 2010;4(10):e859. doi: 10.1371/journal.pntd.0000859 21049013 PMC2964302

[7] de Araújo VE, Pinheiro LC, Almeida MC, de Menezes FC, Morais MH, Reis IA, et al. Relative risk of visceral leishmaniasis in Brazil: a spatial analysis in urban area. PLoS Negl Trop Dis. 2013;7(11):e2540. doi: 10.1371/journal.pntd.0002540 24244776 PMC3820760

[8] Gomes YM, Paiva Cavalcanti M, Lira RA, Abath FG, Alves LC. Diagnosis of canine visceral leishmaniasis: biotechnological advances. Vet J. 2008;175(1):45–52. doi: 10.1016/j.tvjl.2006.10.019 17150389

[9] Herwaldt BL. Leishmaniasis. Lancet. 1999;354(9185):1191–9. doi: 10.1016/S0140-6736(98)10178-2 10513726

[10] Galvão-Castro B, Sá Ferreira JA, Marzochi KF, Marzochi MC, Coutinho SG, Lambert PH. et.al Polyclonal B cell activation, circulating immune complexes and autoimmunity in human american visceral leishmaniasis. Clin Exp Immunol. 1984;56(1):58–66. 6424987 PMC1535977

[11] Singh S. New developments in diagnosis of leishmaniasis. Indian J Med Res. 2006;123(3):311–30. 16778313

[12] Boelaert M, Verdonck K, Menten J, Sunyoto T, van Griensven J, Chappuis F, et al. Rapid tests for the diagnosis of visceral leishmaniasis in patients with suspected disease. Cochrane Database Syst Rev. 2014(6):CD009135. doi: 10.1002/14651858.CD009135.pub2 24947503 PMC4468926

[13] Miró G, Cardoso L, Pennisi MG, Oliva G, Baneth G. Canine leishmaniosis—new concepts and insights on an expanding zoonosis: part two. Trends Parasitol. 2008;24(8):371–7. doi: 10.1016/j.pt.2008.05.003 18603476

[14] Solano-Gallego L, Villanueva-Saz S, Carbonell M, Trotta M, Furlanello T, Natale A. et.al Serological diagnosis of canine leishmaniosis: comparison of three commercial ELISA tests (Leiscan, ID Screen and Leishmania 96), a rapid test (Speed Leish K) and an in-house IFAT. Parasit Vectors. 2014;7:111. doi: 10.1186/1756-3305-7-111 24655335 PMC3994334

[15] Vallur AC, Reinhart C, Mohamath R, Goto Y, Ghosh P, Mondal D, et al. Accurate Serodetection of Asymptomatic Leishmania donovani Infection by Use of Defined Antigens. J Clin Microbiol. 2016;54(4):1025–30. doi: 10.1128/JCM.02620-15 26842701 PMC4809943

[16] Faria AR, de Castro Veloso L, Coura-Vital W, Reis AB, Damasceno LM, Gazzinelli RT, et al. Novel recombinant multiepitope proteins for the diagnosis of asymptomatic leishmania infantum-infected dogs. PLoS Negl Trop Dis. 2015;9(1):e3429. doi: 10.1371/journal.pntd.0003429 25569685 PMC4287523

[17] Celeste BJ, Arroyo Sanchez MC, Ramos-Sanchez EM, Castro LGM, Lima Costa FA, Goto H. et.al Recombinant Leishmania infantum heat shock protein 83 for the serodiagnosis of cutaneous, mucosal, and visceral leishmaniases. Am J Trop Med Hyg. 2014;90(5):860–5. doi: 10.4269/ajtmh.13-0623 24615136 PMC4015579

[18] Farahmand M, Nahrevanian H. Application of Recombinant Proteins for Serodiagnosis of Visceral Leishmaniasis in Humans and Dogs. Iran Biomed J. 2016;20(3):128–34. doi: 10.7508/ibj.2016.03.001 26883952 PMC4949976

[19] Bhattacharyya T, Marlais T, Miles MA. Diagnostic antigens for visceral leishmaniasis: clarification of nomenclatures. Parasit Vectors. 2017;10(1):178. doi: 10.1186/s13071-017-2120-x 28407812 PMC5390433

[20] Goto Y, Coler RN, Guderian J, Mohamath R, Reed SG. Cloning, characterization, and serodiagnostic evaluation of Leishmania infantum tandem repeat proteins. Infect Immun. 2006;74(7):3939–45. doi: 10.1128/IAI.00101-06 16790767 PMC1489730

[21] Stahl HD, Crewther PE, Anders RF, Brown GV, Coppel RL, Bianco AE, et al. Interspersed blocks of repetitive and charged amino acids in a dominant immunogen of Plasmodium falciparum. Proc Natl Acad Sci U S A. 1985;82(2):543–7. doi: 10.1073/pnas.82.2.543 3881769 PMC397076

[22] Davies HM, Nofal SD, McLaughlin EJ, Osborne AR. Repetitive sequences in malaria parasite proteins. FEMS Microbiol Rev. 2017;41(6):923–40. doi: 10.1093/femsre/fux046 29077880 PMC5812512

[23] Burns JM, Shreffler WG, Benson DR, Ghalib HW, Badaro R, Reed SG. et.al Molecular characterization of a kinesin-related antigen of Leishmania chagasi that detects specific antibody in African and American visceral leishmaniasis. Proc Natl Acad Sci U S A. 1993;90(2):775–9. doi: 10.1073/pnas.90.2.775 8421715 PMC45748

[24] Ghedin E, Zhang WW, Charest H, Sundar S, Kenney RT, Matlashewski G. Antibody response against a Leishmania donovani amastigote-stage-specific protein in patients with visceral leishmaniasis. Clin Diagn Lab Immunol. 1997;4(5):530–5. doi: 10.1128/cdli.4.5.530-535.1997 9302200 PMC170587

[25] Bhatia A, Daifalla NS, Jen S, Badaro R, Reed SG, Skeiky YA. et.al Cloning, characterization and serological evaluation of K9 and K26: two related hydrophilic antigens of Leishmania chagasi. Mol Biochem Parasitol. 1999;102(2):249–61. doi: 10.1016/s0166-6851(99)00098-5 10498181

[26] Dhom-Lemos L, Viana AG, Cunha JLR, Cardoso MS, Mendes TAO, Pinheiro GRG, et al. Leishmania infantum recombinant kinesin degenerated derived repeat (rKDDR): A novel potential antigen for serodiagnosis of visceral leishmaniasis. PLoS One. 2019;14(1):e0211719. doi: 10.1371/journal.pone.0211719 30703138 PMC6355020

[27] Palatnik-de-Sousa CB, Batista-de-Melo LM, Borja-Cabrera GP, Palatnik M, Lavor CC. Improving methods for epidemiological control of canine visceral leishmaniasis based on a mathematical model. Impact on the incidence of the canine and human disease. An Acad Bras Cienc. 2004;76(3):583–93. doi: 10.1590/s0001-37652004000300012 15334256

[28] Costa MM, Penido M, dos Santos MS, Doro D, de Freitas E, Michalick MS, et al. Improved canine and human visceral leishmaniasis immunodiagnosis using combinations of synthetic peptides in enzyme-linked immunosorbent assay. PLoS Negl Trop Dis. 2012;6(5):e1622. doi: 10.1371/journal.pntd.0001622 22629475 PMC3358334

[29] Zijlstra EE, Nur Y, Desjeux P, Khalil EA, El-Hassan AM, Groen J. et.al Diagnosing visceral leishmaniasis with the recombinant K39 strip test: experience from the Sudan. Trop Med Int Health. 2001;6(2):108–13. doi: 10.1046/j.1365-3156.2001.00680.x 11251906

[30] Kühne V, Rezaei Z, Pitzinger P, Büscher P. Systematic review on antigens for serodiagnosis of visceral leishmaniasis, with a focus on East Africa. PLoS Negl Trop Dis. 2019;13(8):e0007658. doi: 10.1371/journal.pntd.0007658 31415564 PMC6711545

[31] Goto Y, Coler RN, Reed SG. Bioinformatic identification of tandem repeat antigens of the Leishmania donovani complex. Infect Immun. 2007;75(2):846–51. doi: 10.1128/IAI.01205-06 17088350 PMC1828517

[32] Goto Y, Carter D, Guderian J, Inoue N, Kawazu S, Reed SG. et.al Upregulated expression of B-cell antigen family tandem repeat proteins by Leishmania amastigotes. Infect Immun. 2010;78(5):2138–45. doi: 10.1128/IAI.01102-09 20160013 PMC2863543

[33] Laemmli UK. Cleavage of structural proteins during the assembly of the head of bacteriophage T4. Nature. 1970;227(5259):680–5. doi: 10.1038/227680a0 5432063

[34] Larsen JE, Lund O, Nielsen M. Improved method for predicting linear B-cell epitopes. Immunome Res. 2006;2:2. doi: 10.1186/1745-7580-2-2 16635264 PMC1479323

[35] Dosztányi Z, Csizmok V, Tompa P, Simon I. IUPred: web server for the prediction of intrinsically unstructured regions of proteins based on estimated energy content. Bioinformatics. 2005;21(16):3433–4. doi: 10.1093/bioinformatics/bti541 15955779

[36] Mary C, Faraut F, Lascombe L, Dumon H. Quantification of Leishmania infantum DNA by a real-time PCR assay with high sensitivity. J Clin Microbiol. 2004;42(11):5249–55. doi: 10.1128/JCM.42.11.5249-5255.2004 15528722 PMC525214

[37] Singh OP, Sundar S. Developments in Diagnosis of Visceral Leishmaniasis in the Elimination Era. J Parasitol Res. 2015;2015:239469. doi: 10.1155/2015/239469 26843964 PMC4710934

[38] Magalhães FB, Castro Neto AL, Nascimento MB, Santos WJT, Medeiros ZM, Lima Neto AS, et al. Evaluation of a new set of recombinant antigens for the serological diagnosis of human and canine visceral leishmaniasis. PLoS One. 2017;12(9):e0184867. doi: 10.1371/journal.pone.0184867 28957332 PMC5619722

[39] Daprà F, Scalone A, Mignone W, Ferroglio E, Mannelli A, Biglino A, et al. Validation of a recombinant based antibody ELISA for diagnosis of human and canine leishmaniasis. J Immunoassay Immunochem. 2008;29(3):244–56. doi: 10.1080/15321810802116006 18569373

[40] Siripattanapipong S, Kato H, Tan-Ariya P, Mungthin M, Leelayoova S. Comparison of Recombinant Proteins of Kinesin 39, Heat Shock Protein 70, Heat Shock Protein 83, and Glycoprotein 63 for Antibody Detection of Leishmania martiniquensis Infection. J Eukaryot Microbiol. 2017;64(6):820–8. doi: 10.1111/jeu.12415 28370779

[41] Chauhan IS, Shukla R, Krishna S, Sekhri S, Kaushik U, Baby S, et al. Recombinant Leishmania Rab6 (rLdRab6) is recognized by sera from visceral leishmaniasis patients. Exp Parasitol. 2016;170:135–47. doi: 10.1016/j.exppara.2016.09.010 27666959

[42] Travi BL, Cordeiro-da-Silva A, Dantas-Torres F, Miró G. Canine visceral leishmaniasis: Diagnosis and management of the reservoir living among us. PLoS Negl Trop Dis. 2018;12(1):e0006082. doi: 10.1371/journal.pntd.0006082 29324838 PMC5764232

[43] Rosati S, Ortoffi M, Profiti M, Mannelli A, Mignone W, Bollo E, et al. Prokaryotic expression and antigenic characterization of three recombinant Leishmania antigens for serological diagnosis of canine leishmaniasis. Clin Diagn Lab Immunol. 2003;10(6):1153–6. doi: 10.1128/cdli.10.6.1153-1156.2003 14607883 PMC262443

[44] Fraga DB, da Silva ED, Pacheco LV, Borja LS, de Oliveira IQ, Coura-Vital W, et al. A multicentric evaluation of the recombinant Leishmania infantum antigen-based immunochromatographic assay for the serodiagnosis of canine visceral leishmaniasis. Parasit Vectors. 2014;7:136. doi: 10.1186/1756-3305-7-136 24684857 PMC3972511

[45] Alves AS, Mouta-Confort E, Figueiredo FB, Oliveira RV, Schubach AO, Madeira MF. et.al Evaluation of serological cross-reactivity between canine visceral leishmaniasis and natural infection by Trypanosoma caninum. Res Vet Sci. 2012;93(3):1329–33. doi: 10.1016/j.rvsc.2012.07.006 22840335

[46] Depledge DP, Dalby AR. COPASAAR—a database for proteomic analysis of single amino acid repeats. BMC Bioinformatics. 2005;6:196. doi: 10.1186/1471-2105-6-196 16078990 PMC1199582

[47] Depledge DP, Lower RP, Smith DF. RepSeq—a database of amino acid repeats present in lower eukaryotic pathogens. BMC Bioinformatics. 2007;8:122. doi: 10.1186/1471-2105-8-122 17428323 PMC1854910

[48] Goto H, Lindoso JA. Current diagnosis and treatment of cutaneous and mucocutaneous leishmaniasis. Expert Rev Anti Infect Ther. 2010;8(4):419–33. doi: 10.1586/eri.10.19 20377337

[49] Coppel RL, Cowman AF, Anders RF, Bianco AE, Saint RB, Lingelbach KR, et al. Immune sera recognize on erythrocytes Plasmodium falciparum antigen composed of repeated amino acid sequences. Nature. 1984;310(5980):789–92. doi: 10.1038/310789a0 6382025

[50] Thuy NT, Goto Y, Lun ZR, Kawazu S, Inoue N. Tandem repeat protein as potential diagnostic antigen for Trypanosoma evansi infection. Parasitol Res. 2012;110(2):733–9. doi: 10.1007/s00436-011-2632-9 21927872

[51] Kemp DJ, Coppel RL, Anders RF. Repetitive proteins and genes of malaria. Annu Rev Microbiol. 1987;41:181–208. doi: 10.1146/annurev.mi.41.100187.001145 3318667

[52] Berzofsky JA. Intrinsic and extrinsic factors in protein antigenic structure. Science. 1985;229(4717):932–40. doi: 10.1126/science.2410982 2410982

[53] Goto Y, Carter D, Reed SG. Immunological dominance of Trypanosoma cruzi tandem repeat proteins. Infect Immun. 2008;76(9):3967–74. doi: 10.1128/IAI.00604-08 18625739 PMC2519453

[54] Sundar S, Singh RK, Bimal SK, Gidwani K, Mishra A, Maurya R, et al. Comparative evaluation of parasitology and serological tests in the diagnosis of visceral leishmaniasis in India: a phase III diagnostic accuracy study. Trop Med Int Health. 2007;12(2):284–9. doi: 10.1111/j.1365-3156.2006.01775.x 17300637

[55] Mohapatra TM, Singh DP, Sen MR, Bharti K, Sundar S. Compararative evaluation of rK9, rK26 and rK39 antigens in the serodiagnosis of Indian visceral leishmaniasis. J Infect Dev Ctries. 2010;4(2):114–7. doi: 10.3855/jidc.544 20212344

[56] Pattabhi S, Whittle J, Mohamath R, El-Safi S, Moulton GG, Guderian JA, et al. Design, development and evaluation of rK28-based point-of-care tests for improving rapid diagnosis of visceral leishmaniasis. PLoS Negl Trop Dis. 2010;4(9). doi: 10.1371/journal.pntd.0000822 20856856 PMC2939046

[57] Terán-Ángel G, Rodríguez V, Silva R, Zerpa O, Schallig H, Ulrich M, et al. Non invasive diagnostic tools for visceral leishmaniasis: a comparison of the immunoserological tests DAT, rK26 and rK39. Biomedica. 2010;30(1):39–45. 20890548

[58] Carvalho SF, Lemos EM, Corey R, Dietze R. Performance of recombinant K39 antigen in the diagnosis of Brazilian visceral leishmaniasis. Am J Trop Med Hyg. 2003;68(3):321–4. 12685638

[59] Chappuis F, Rijal S, Soto A, Menten J, Boelaert M. A meta-analysis of the diagnostic performance of the direct agglutination test and rK39 dipstick for visceral leishmaniasis. BMJ. 2006;333(7571):723. doi: 10.1136/bmj.38917.503056.7C 16882683 PMC1592383

[60] Abass E, Bollig N, Reinhard K, Camara B, Mansour D, Visekruna A, et al. rKLO8, a novel Leishmania donovani—derived recombinant immunodominant protein for sensitive detection of visceral leishmaniasis in Sudan. PLoS Negl Trop Dis. 2013;7(7):e2322. doi: 10.1371/journal.pntd.0002322 23875052 PMC3715527

[61] Abass E, Kang C, Martinkovic F, Semião-Santos SJ, Sundar S, Walden P, et al. Heterogeneity of Leishmania donovani parasites complicates diagnosis of visceral leishmaniasis: comparison of different serological tests in three endemic regions. PLoS One. 2015;10(3):e0116408. doi: 10.1371/journal.pone.0116408 25734336 PMC4348478

[62] Peruhype-Magalhães V, Machado-de-Assis TS, Rabello A. Use of the Kala-Azar Detect and IT-LEISH rapid tests for the diagnosis of visceral leishmaniasis in Brazil. Mem Inst Oswaldo Cruz. 2012;107(7):951–2. doi: 10.1590/s0074-02762012000700019 23147155

[63] Machado de Assis TS, Rabello A, Werneck GL. Latent class analysis of diagnostic tests for visceral leishmaniasis in Brazil. Trop Med Int Health. 2012;17(10):1202–7. doi: 10.1111/j.1365-3156.2012.03064.x 22897740

