# Supplementary figures and images for: The increased presence of repetitive motifs in the KDDR-plus recombinant protein, a kinesin-derived antigen from Leishmania infantum, improves the diagnostic performance of serological tests for human and canine visceral leishmaniasis
Source: PLoS Negl Trop Dis. 2021 Sep 17;15(9):e0009759. doi: 10.1371/journal.pntd.0009759 (PMC8480608; doi:10.1371/journal.pntd.0009759)

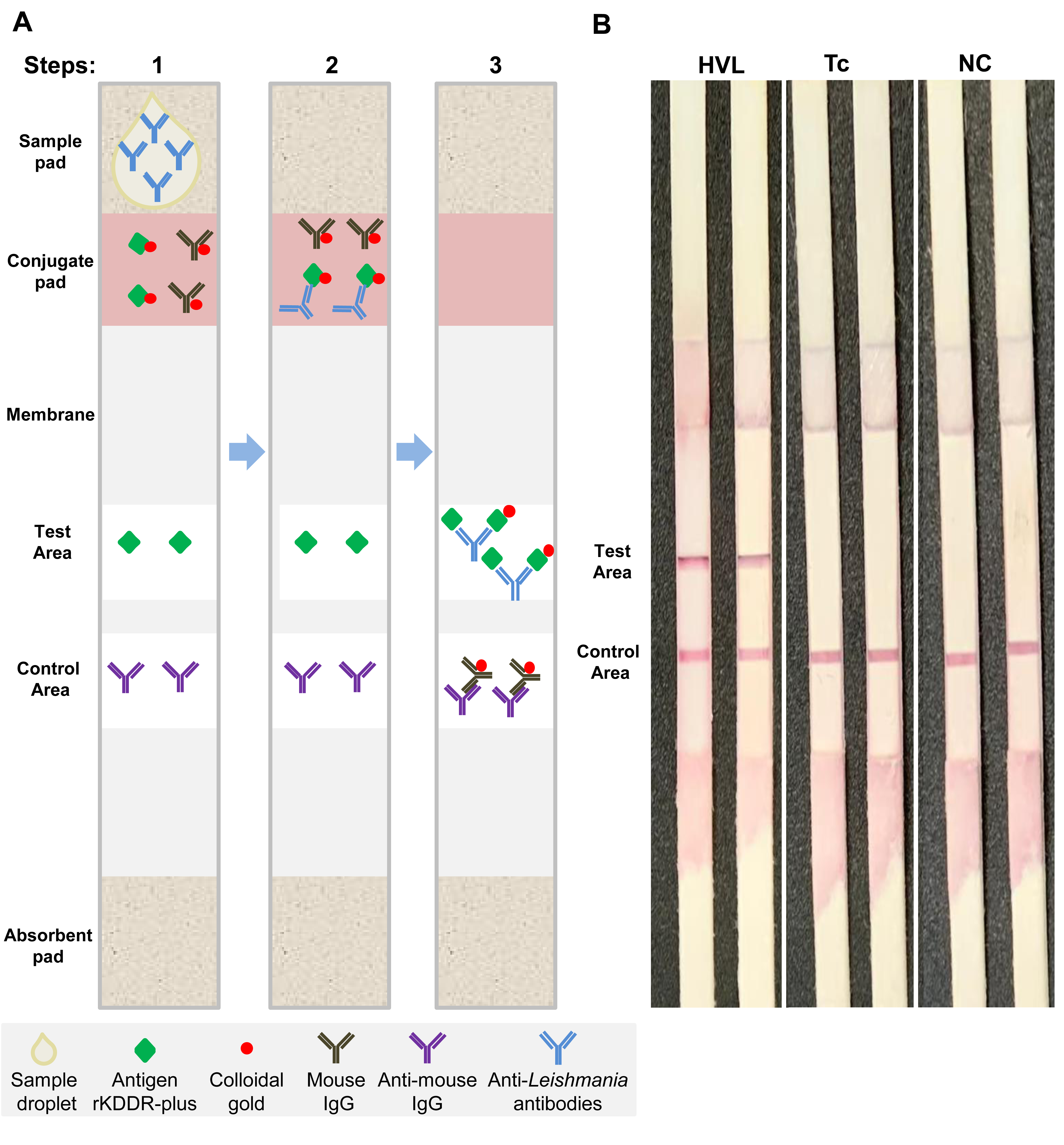

Supplement: S1 Fig — (A) Schematic representation of the diagnostic ICT device. The three schematic strips represent the test steps: 1) sample application; 2) sample migration; 3) test result. (B) Photograph of representative results of ICT/rKDDR-plus strips tested with different human serum samples. HVL: test with sera from two patients infected with Leishmania; Tc: test with sera from two patients infected with T. cruzi; NC: test with two negative sera from healthy individuals. With negative serum samples for leishmaniasis (NC and Tc), only the control line is present, while with serum samples from VL patients, both control and test lines turned red. (TIF) [file pntd.0009759.s001.tif]

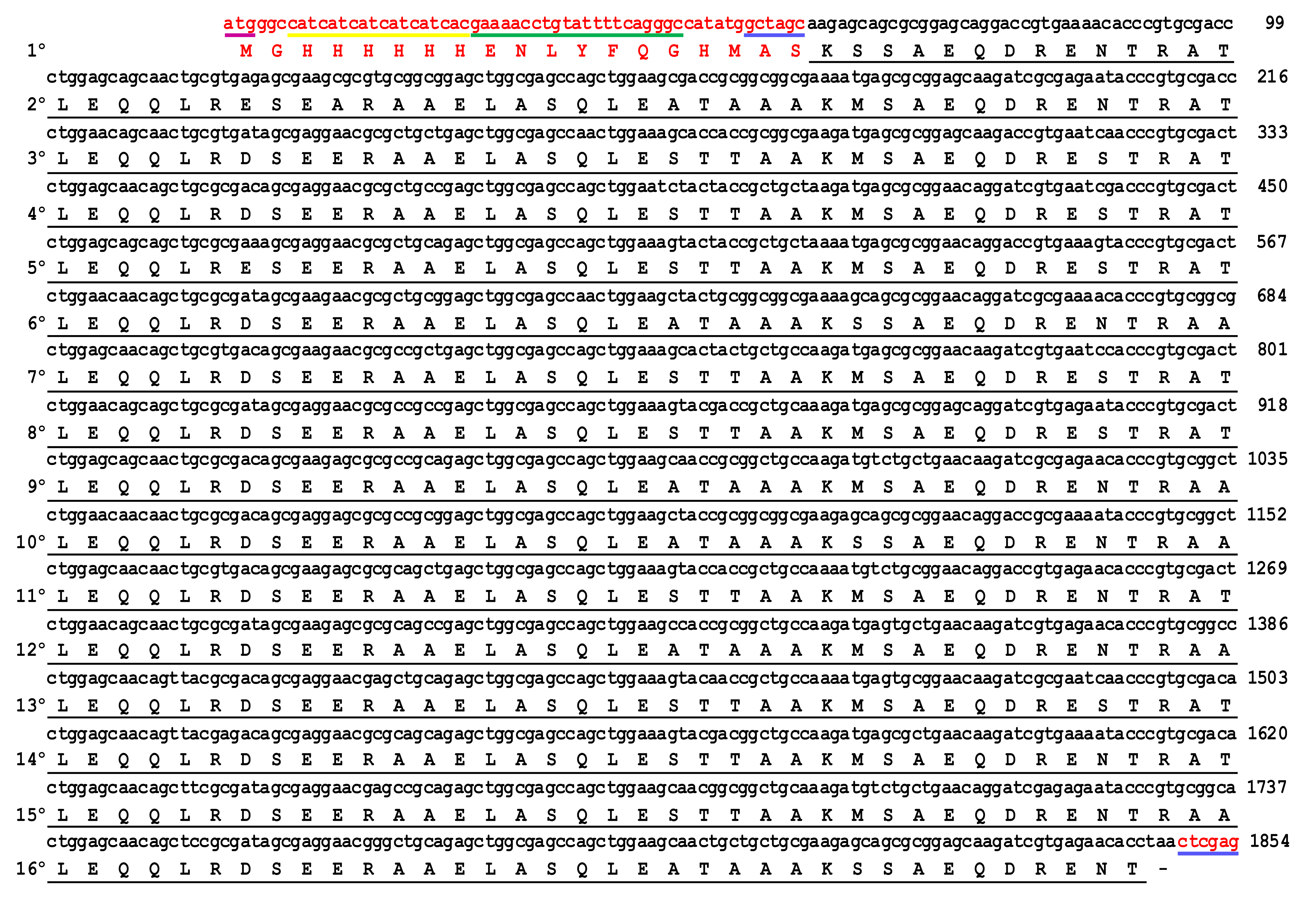

Supplement: S2 Fig — Lowercase letters represent the nucleotide sequence of KDDR-plus and uppercase letters represent the translated protein sequence. The nucleotides and amino acids indicated in red at the ends of the sequences represent the portion derived from the plasmid pET28a-TEV, used in the expression of the protein in bacteria. The underlined nucleotides in purple correspond to the initiation codon; the underlined in yellow represent the histidine tag added to the protein to facilitate the purification process; the underlined nucleotides in blue correspond to the sites for the restriction enzymes; and the underlined in green represent the cleavage site for the TEV protease. The remainder of the underlined sequence shows the 15.3 repetitive motifs of 39 amino acids derived from the L. infantum kinesin protein, except for the first motif with 14 amino acids and the 16th motif with 36 amino acids. The ordinal numbers indicated on the left represent the number of repetitive motifs of rKDDR-plus. The numbers on the right represent the position of the nucleotides. (TIF) [file pntd.0009759.s002.tif]

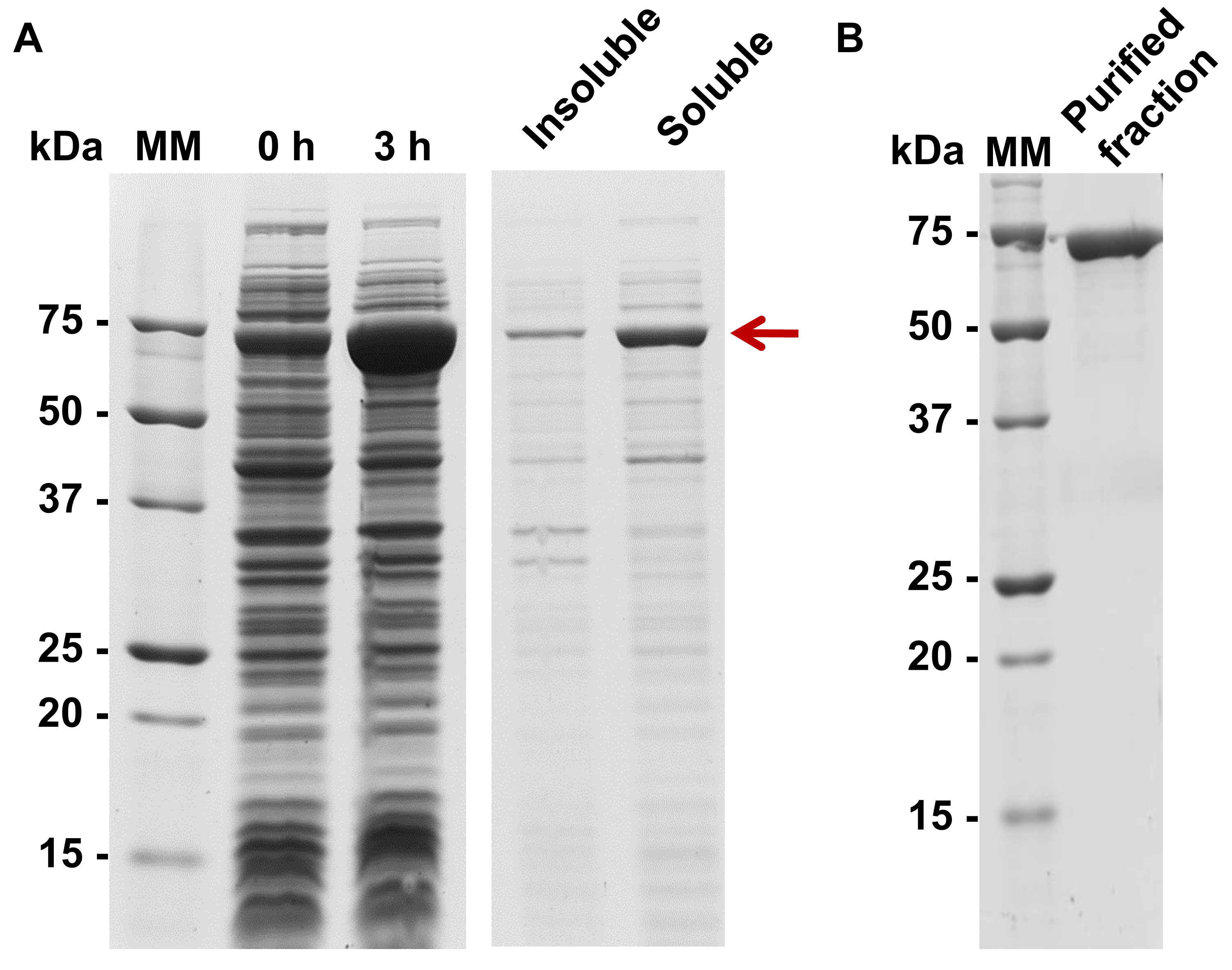

Supplement: S3 Fig — (A) Extracts of E. coli bacteria, BL21 Star strain, containing the plasmid pET28a-TEV/KDDR-plus, before (0h) and after (3h) induction of the recombinant protein with IPTG (1 mM). The bacterial extract was lysed and separated into soluble and insoluble fraction by centrifugation. The red arrow indicates the rKDDR-plus protein band after expression and solubility test. (B) After purification by affinity chromatography of the soluble fraction of the bacterial lysate, the purified fraction presented a band of approximately 68 kDa, corresponding to rKDDR-plus. MM: molecular mass marker; kDa: kilodalton. (TIF) [file pntd.0009759.s003.tif]

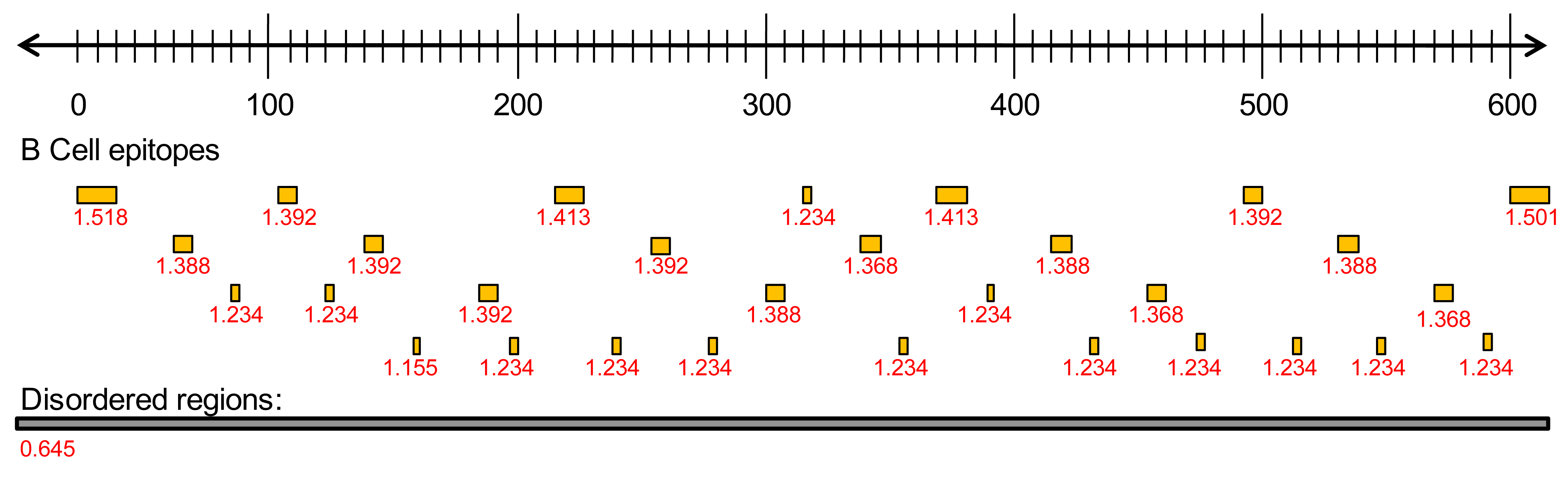

Supplement: S4 Fig — The dashed arrow corresponds to the complete amino acid sequence of the protein. The orange boxes correspond to the linear B-cell epitopes predicted by the BepiPred program, while the gray box corresponds to the prediction of structural protein disorder using the IUPred program. The value below each box corresponds to the score of each prediction. (TIF) [file pntd.0009759.s004.tif]
