# Supplementary material for: The increased presence of repetitive motifs in the KDDR-plus recombinant protein, a kinesin-derived antigen from Leishmania infantum, improves the diagnostic performance of serological tests for human and canine visceral leishmaniasis
Source: PLoS Negl Trop Dis. 2021 Sep 17;15(9):e0009759. doi: 10.1371/journal.pntd.0009759 (PMC8480608; doi:10.1371/journal.pntd.0009759)
Supplement: S1 File — Data used to make ELISA figures and tables. (PDF) [file pntd.0009759.s005.pdf]

Patients infected with *Leishmania* (HVL)					
Antigens	KDDR-PLUS	KDDR	K39	K26	Crude Soluble Antigen
Cut-off	**0,3611**	**0,4437**	**0,5839**	**0,3977**	**1,2840**
ID	Absorbances				
**HVL 01**	3,0964	2,7780	3,1044	1,0847	3,1816
**HVL 02**	3,6074	3,4267	3,5352	0,5593	3,0246
**HVL 03**	3,4426	3,3502	3,3837	0,1099	1,7494
**HVL 04**	3,6532	3,4313	3,5938	3,4988	3,3503
**HVL 05**	0,7088	0,7350	0,6530	0,1348	3,3211
**HVL 06**	1,2011	1,4265	1,3042	1,6012	3,3661
**HVL 07**	3,6041	3,4179	3,4918	2,0865	3,3519
**HVL 08**	1,9221	1,9433	1,5976	0,3341	2,4003
**HVL 09**	0,5955	0,4007	0,8525	0,1592	2,8857
**HVL 10**	0,3711	0,4550	0,3931	0,4496	1,5249
**HVL 11**	3,4370	3,4196	3,4845	1,9561	3,2996
**HVL 12**	3,4747	3,4672	3,4857	0,2404	2,8493
**HVL 13**	0,5244	0,6238	0,7152	1,4913	2,7634
**HVL 14**	3,1134	2,9627	3,2204	2,2766	3,2256
**HVL 15**	1,6741	2,0231	1,8979	0,1964	2,8984
**HVL 16**	3,6233	3,5673	3,5693	3,5917	3,3651
**HVL 17**	3,3038	3,2067	3,3722	0,3386	3,3595
**HVL 18**	0,4836	0,5486	0,6696	0,2746	2,4318
**HVL 19**	3,4659	3,4391	3,4880	3,4543	3,2643
**HVL 20**	3,4390	3,4187	3,4664	1,4632	3,3088
**HVL 21**	3,3545	3,3209	3,3887	2,9307	3,2943
**HVL 22**	3,1082	3,0794	3,2186	0,9493	3,2439
**HVL 23**	3,5095	3,4521	3,5643	1,2935	3,2788
**HVL 24**	3,1019	2,9761	3,3511	2,6792	3,2595
**HVL 25**	3,5086	3,4502	3,6102	1,3481	3,3765
**HVL 26**	3,5099	3,4620	3,6066	1,2789	3,3755
**HVL 27**	1,7326	1,0007	2,5968	1,7967	3,2914
**HVL 28**	2,9593	2,2055	3,2254	1,2832	3,2683
**HVL 29**	3,2402	3,1513	3,3784	2,9340	3,1803
**HVL 30**	3,5833	3,4117	3,5015	1,7980	3,3167
**HVL 31**	3,5258	3,3268	3,4374	0,6988	3,2434
**HVL 32**	3,6517	3,4399	3,6571	1,3512	3,2784
**HVL 33**	3,6801	3,4691	3,5734	0,7070	3,2947
**HVL 34**	3,6461	3,3565	3,5533	3,2152	3,3358
**HVL 35**	3,3482	3,0147	3,2447	1,0926	3,3045
**HVL 36**	0,2855	0,4437	0,7370	2,0291	3,1709
**HVL 37**	2,8938	2,4545	2,9046	1,0280	3,2953
**HVL 38**	3,5483	3,5447	3,6137	1,1602	3,3652
**HVL 39**	3,4353	3,5545	3,5053	2,1793	3,3232
**HVL 40**	3,5146	3,5710	3,5501	2,8692	3,2944
**HVL 41**	3,4792	3,4394	3,4799	3,2660	3,2767
**HVL 42**	3,4866	3,4550	3,4690	2,6284	3,3433
**HVL 43**	2,9122	2,1194	3,0789	2,5836	3,1925
**HVL 44**	3,4337	3,4211	3,5641	0,8403	3,3262
**HVL 45**	3,4443	3,3833	3,5425	1,2644	3,1751
**HVL 46**	3,3722	3,3482	3,4429	1,3516	3,3551
**HVL 47**	3,2473	3,2939	3,3960	1,9159	3,2727
**HVL 48**	3,5079	3,4697	3,6195	1,1512	1,6695
**HVL 49**	3,5002	3,4160	3,5719	0,3459	3,3905
**HVL 50**	3,4697	3,4555	3,5537	2,5033	3,3641

Healthy individuals					
Antigens	KDDR-PLUS	KDDR	K39	K26	Crude Soluble Antigen
Cut-off	0,3611	0,4437	0,5839	0,3977	1,2840
ID	Absorbances				
NC 01	0,2791	0,2695	0,3405	0,0676	0,2784
NC 02	0,1075	0,1162	0,2909	0,0477	0,4347
NC 03	0,1062	0,2784	0,2274	0,0803	0,4437
NC 04	0,1310	0,1157	0,2891	0,3640	0,4996
NC 05	0,3013	0,2630	0,4680	0,2348	0,2620
NC 06	0,3013	0,2560	0,3427	0,1287	0,2353
NC 07	0,2797	0,2044	0,3929	0,1747	0,2933
NC 08	0,1091	0,2898	0,3017	0,0857	0,2503
NC 09	0,2517	0,2720	0,2444	0,1610	0,3203
NC 10	0,1153	0,2330	0,2585	0,0976	0,3336
NC 11	0,0344	0,1558	0,1729	0,0833	0,3142
NC 12	0,3512	0,2191	0,6121	0,1319	0,0110
NC 13	0,0963	0,3089	0,3499	0,1370	0,4674
NC 14	0,1290	0,2579	0,5994	0,1424	0,4805
NC 15	0,0551	0,1493	0,1992	0,0909	0,9298
NC 16	0,1160	0,2024	0,1863	0,2317	0,6623
NC 17	0,0676	0,1646	0,2069	0,1027	0,1550
NC 18	0,1279	0,2455	0,4279	0,1401	0,3161
NC 19	0,0460	0,1304	0,4009	0,1618	0,0569
NC 20	0,0735	0,2385	0,2701	0,2309	0,6232
NC 21	0,1065	0,1093	0,2794	0,4331	0,1306
NC 22	0,2116	0,2348	0,4881	0,4423	1,0429

Patients infected with *T. cruzi*					
**Antigens**	**KDDR-PLUS**	**KDDR**	**K39**	**K26**	**Crude Soluble Antigen**
**Cut-off**	**0,3611**	**0,4437**	**0,5839**	**0,3977**	**1,2840**
**ID**	**Absorbances**				
**Tc 01**	0,1885	0,2844	0,7727	0,2477	3,2810
**Tc 02**	0,1950	0,2029	0,3077	0,1335	3,1596
**Tc 03**	0,1988	0,3051	0,6811	0,1942	3,2997
**Tc 04**	0,3248	0,3622	0,4980	0,2514	3,3608
**Tc 05**	0,3198	0,3112	1,0202	0,4473	2,7374
**Tc 06**	0,2677	0,2186	0,3838	0,1149	3,1639
**Tc 07**	0,1633	0,2707	0,3533	0,4440	3,1885
**Tc 08**	0,2204	0,2653	0,7973	0,9385	3,2243
**Tc 09**	0,1176	0,1673	0,8147	0,6162	3,1710
**Tc 10**	0,3185	0,3085	0,4966	0,1708	3,2660
**Tc 11**	0,2875	0,3221	1,2015	0,5739	3,1935
**Tc 12**	0,0726	0,0848	0,1018	0,0473	1,7341
**Tc 13**	0,1045	0,1212	0,1739	0,0727	3,3109
**Tc 14**	0,3271	0,2585	1,1206	1,7427	3,3094
**Tc 15**	0,2143	0,4436	0,4157	0,4053	3,1162
**Tc 16**	0,0585	0,1319	0,5431	0,0872	2,6317
**Tc 17**	0,2703	0,3940	0,5404	0,2216	3,0359
**Tc 18**	0,2356	0,2719	0,5529	0,3999	3,2650
**Tc 19**	0,0798	0,1771	0,1822	0,0943	1,8765
**Tc 20**	0,1221	0,2229	2,1458	0,3391	3,2795
**Tc 21**	0,1825	0,2981	0,9816	0,2396	3,3108
**Tc 22**	0,2112	0,2654	0,7307	0,3956	2,6696
**Tc 23**	0,1198	0,2864	0,3396	0,3323	3,3311
**Tc 24**	0,1281	0,2116	1,2048	0,3148	3,3117
**Tc 25**	0,1064	0,4438	0,2558	0,1421	3,3357
**Tc 26**	0,0699	0,2044	0,1944	0,1631	0,5092
**Tc 27**	0,2313	0,4592	2,4565	0,1619	3,3638
**Tc 28**	0,1898	0,3401	0,4548	0,5644	3,2590
**Tc 29**	0,1071	0,2545	0,4357	0,1410	3,2379
**Tc 30**	0,1794	0,2290	0,3852	0,1428	3,2769
**Tc 31**	0,0997	0,2225	0,3181	0,2417	3,2331
**Tc 32**	0,1693	0,3543	0,6111	0,2109	3,0798
**Tc 33**	0,0682	0,3567	0,2529	0,0695	3,3341
**Tc 34**	0,0921	0,2282	0,3455	0,1862	3,1564
**Tc 35**	0,0818	0,1791	0,1813	0,0853	3,3569
**Tc 36**	0,1777	0,4375	0,2886	0,2071	3,3917
**Tc 37**	0,1617	0,3106	1,1289	0,4946	3,2346
**Tc 38**	0,1799	0,2602	2,2326	0,2013	3,4596
**Tc 39**	0,1724	0,2208	0,4518	0,1563	3,2567
**Tc 40**	0,2540	0,3792	0,7782	0,1408	3,3022
**Tc 41**	0,0657	0,1594	0,8732	0,1283	3,2578
**Tc 42**	0,1388	0,1777	0,3877	0,1106	3,3694
**Tc 43**	0,1633	0,2407	0,4899	0,3157	3,4150
**Tc 44**	0,1292	0,4904	1,5769	0,1264	3,3237
**Tc 45**	0,1080	0,1258	0,3634	0,3377	3,0889
**Tc 46**	0,2837	0,4039	0,7957	0,3386	2,9035
**Tc 47**	0,1264	0,3292	0,5684	0,4707	3,3198
**Tc 48**	0,1280	0,1743	0,1965	0,1658	2,6465
**Tc 49**	0,1103	0,2812	0,1653	0,0604	3,3431
**Tc 50**	0,1735	0,2893	0,4847	0,5914	3,2315
**Tc 51**	0,0657	0,1582	0,2478	0,2396	2,0818
**Tc 52**	0,1457	0,3242	0,2683	0,2427	2,7332
**Tc 53**	0,1546	0,2119	0,7163	0,8370	3,0576
**Tc 54**	0,1342	0,2169	0,3556	0,1539	2,3370

Dogs infected with *Leishmania* (CVL)					
Antigens	KDDR-PLUS	KDDR	K39	K26	Crude Soluble Antigen
Cut-off	0,4087	0,3248	0,3147	0,3251	0,8314
ID	Absorbances				
CVL 01	2,1148	2,1445	2,2975	1,5439	1,7236
CVL 02	0,6487	0,8505	0,5230	0,6174	1,4507
CVL 03	2,4962	2,3383	2,5758	0,9097	1,3731
CVL 04	2,7717	2,4908	2,6302	1,3963	1,7615
CVL 05	2,1730	1,9944	2,1499	0,8497	1,7279
CVL 06	2,6162	2,2459	2,4402	0,9849	1,5017
CVL 07	2,7507	2,6108	2,7502	1,5559	1,6503
CVL 08	1,3003	1,3484	1,5344	0,9487	1,5768
CVL 09	2,0108	1,9020	2,1713	0,6510	1,3884
CVL 10	2,2287	2,1835	2,3709	1,8312	1,5856
CVL 11	2,2114	2,1351	2,2763	1,6170	1,4986
CVL 12	2,7231	2,4433	2,6379	1,5070	1,4408
CVL 13	1,3910	1,5261	1,7966	1,3598	1,4811
CVL 14	2,4476	2,3982	2,4255	1,0183	1,6880
CVL 15	2,7751	2,3600	2,5517	1,3970	1,1911
CVL 16	1,6625	2,0561	2,0827	0,9597	1,7315
CVL 17	1,9362	2,0585	2,1240	1,6289	1,6855
CVL 18	2,4900	2,3979	2,5325	1,9082	1,7371
CVL 19	2,1560	2,0490	1,9989	2,0728	1,7581
CVL 20	2,8291	2,6275	2,7359	0,6118	1,6412
CVL 21	2,5010	2,5573	2,6389	1,1886	1,8999
CVL 22	2,2646	2,1806	2,3788	1,3205	1,6214
CVL 23	0,4604	0,6203	0,5814	0,8248	0,6759
CVL 24	1,7383	1,8448	1,9135	0,9037	0,8566
CVL 25	2,1443	2,0799	2,2030	1,4966	0,9216
CVL 26	0,3833	0,4456	0,4528	0,2538	1,0389
CVL 27	0,7580	0,3850	0,9150	0,2315	0,8038
CVL 28	0,5361	0,5145	0,4484	0,1569	1,1341
CVL 29	0,3354	0,9132	0,2278	0,8213	0,9527
CVL 30	1,1661	0,4801	0,4306	0,6778	1,3459
CVL 31	0,4548	0,4502	0,4210	0,5448	0,9730
CVL 32	0,7816	0,5191	0,4869	0,2518	0,8378
CVL 33	0,6561	0,3846	0,4568	0,3704	1,2345
CVL 34	2,2531	2,4946	2,5673	1,5577	1,6973
CVL 35	0,6553	0,3185	0,5032	0,5611	0,9487
CVL 36	1,7615	2,0559	2,2168	1,3886	1,9265
CVL 37	0,7670	0,5873	0,5609	0,8216	1,7675
CVL 38	0,4197	0,4516	0,5332	1,2931	1,3511
CVL 39	0,5225	0,3871	0,3151	0,6573	1,0572
CVL 40	0,4747	0,2282	0,1563	0,6097	1,0301
CVL 41	0,4820	0,5500	0,3783	0,4480	0,9389
CVL 42	0,4658	0,4452	0,4824	0,3279	1,4532
CVL 43	1,9484	1,9493	2,3176	1,3932	1,7593
CVL 44	0,4122	0,3311	0,3625	0,3274	1,5659
CVL 45	2,2216	2,0167	2,2560	1,5093	1,6353
CVL 46	1,5280	1,1643	1,3738	0,9201	1,0874
CVL 47	1,6032	1,2681	1,5651	0,9045	1,5360
CVL 48	1,4369	1,4273	1,4668	1,2487	1,3348
CVL 49	1,9830	2,0825	2,3610	0,5556	1,6984
CVL 50	1,3144	1,3250	1,5950	0,8595	1,6433
CVL 51	0,5273	0,4388	0,5366	0,5364	1,3837
CVL 52	2,5153	2,1292	2,4157	1,2259	1,5422
CVL 53	0,6567	0,3438	0,4258	0,3725	1,1082
CVL 54	2,3789	2,1643	2,4359	1,0138	1,3109
CVL 55	1,9038	2,1194	2,2574	0,7747	1,9043
CVL 56	1,3074	1,3913	1,4131	0,8451	1,5000
CVL 57	0,4643	0,4814	0,4073	0,2115	1,7548
CVL 58	0,8787	1,0617	0,9187	0,5984	1,9087
CVL 59	1,4480	1,3753	1,3544	0,3862	0,9483
CVL 60	1,6608	1,5266	1,5241	0,4863	1,9624

Non-infected healthy dogs					
Antigens	KDDR-PLUS	KDDR	K39	K26	Crude Soluble Antigen
Cut-off	0,4087	0,3248	0,3147	0,3251	0,8314
ID	Absorbances				
NC 01	0,1267	0,1386	0,1268	0,2170	0,4918
NC 02	0,1800	0,1217	0,1318	0,0653	0,4431
NC 03	0,1985	0,2398	0,1881	0,2900	0,5801
NC 04	0,1887	0,1807	0,0987	0,0598	0,5402
NC 05	0,1003	0,1447	0,0591	0,2438	0,3638
NC 06	0,1408	0,1040	0,0465	0,1152	0,2902
NC 07	0,1497	0,1079	0,0793	0,0407	0,3390
NC 08	0,2992	0,2494	0,1695	0,1734	0,4435
NC 09	0,0765	0,1640	0,0860	0,2314	0,4975
NC 10	0,5603	0,1282	0,1126	0,1265	0,5310
NC 11	0,3339	0,1309	0,1034	0,1602	0,5996
NC 12	0,5479	0,1978	0,2523	0,1067	0,4237
NC 13	0,2418	0,1478	0,1332	0,3464	0,5768
NC 14	0,2081	0,1612	0,1491	0,1740	0,5173
NC 15	0,1778	0,1658	0,1044	0,0991	0,4383
NC 16	0,3154	0,1350	0,0697	0,1473	0,3858
NC 17	0,0837	0,0948	0,0817	0,0702	0,4683
NC 18	0,3932	0,1094	0,0925	0,0454	0,5073
NC 19	0,0890	0,1171	0,0890	0,0266	0,3747
NC 20	0,2779	0,1732	0,1705	0,0383	0,4582
NC 21	0,1632	0,1529	0,1221	0,0271	0,5180
NC 22	0,1836	0,1641	0,1006	0,0529	0,5255
NC 23	0,2642	0,2611	0,2369	0,1116	0,5564
NC 24	0,2186	0,2537	0,2261	0,0957	0,4786
NC 25	0,2184	0,1611	0,1529	0,0802	0,4995
NC 26	0,3104	0,1989	0,1587	0,3804	0,5548
NC 27	0,3371	0,1843	0,2378	0,0898	0,5135
NC 28	0,3210	0,1525	0,1418	0,0790	0,4511
NC 29	0,1689	0,0316	0,1082	0,0785	0,4342
NC 30	0,2090	0,0515	0,2097	0,1021	0,5476
NC 31	0,1743	0,0453	0,1289	0,1286	0,5026
NC 32	0,2450	0,0508	0,2049	0,1035	0,5535
NC 33	0,3254	0,0492	0,0868	0,1511	0,6957
NC 34	0,3156	0,0709	0,1104	0,2703	0,3193
NC 35	0,1344	0,1232	0,1813	0,0757	0,2884
NC 36	0,1282	0,1153	0,1636	0,0719	0,2999

Dogs infected with *T. cruzi*					
**Antigens**	**KDDR-PLUS**	**KDDR**	**K39**	**K26**	**Crude Soluble Antigen**
**Cut-off**	**0,4087**	**0,3248**	**0,3147**	**0,3251**	**0,8314**
**ID**	**Absorbances**				
**Tc 01**	0,3489	0,1779	0,1312	0,1014	0,7261
**Tc 02**	0,0579	0,0109	0,0629	0,0497	0,1421
**Tc 03**	0,0205	0,0583	0,0052	0,0182	0,6288
**Tc 04**	0,2304	0,0935	0,1015	0,1752	1,1012
**Tc 05**	0,0754	0,1357	0,1091	0,0915	0,2822
**Tc 06**	0,3043	0,2015	0,1835	0,2512	0,5987
**Tc 07**	0,3405	0,3549	0,3143	0,1659	0,6139
**Tc 08**	0,5601	0,2967	0,2855	1,2262	1,1070
**Tc 09**	0,5212	0,2321	0,2648	0,7988	1,1382
**Tc 10**	0,2486	0,1623	0,1718	0,0953	0,6336
**Tc 11**	0,1385	0,1384	0,1050	0,1930	0,5628
**Tc 12**	0,2526	0,1947	0,1295	0,1542	0,5939
**Tc 13**	0,2758	0,1580	0,1136	0,0659	1,4444
**Tc 14**	0,3011	0,1096	0,1003	0,0846	0,4772
**Tc 15**	0,2675	0,1666	0,1423	0,5997	1,5117
**Tc 16**	0,2222	0,1465	0,0924	0,2235	1,2731
**Tc 17**	0,2035	0,1900	0,0765	0,2056	0,6183
**Tc 18**	0,1519	0,1012	0,0481	0,0695	0,4676
**Tc 19**	0,1409	0,1413	0,0856	0,0411	0,4927
**Tc 20**	0,3533	0,3517	0,2097	0,3010	1,6019
**Tc 21**	0,5572	0,3433	0,2455	0,0884	1,2765
**Tc 22**	0,4052	0,2216	0,1992	0,1237	0,5501
**Tc 23**	0,6224	0,1590	0,0772	0,0528	1,4193
**Tc 24**	0,2888	0,1853	0,1767	0,1304	1,5280
**Tc 25**	0,3456	0,2807	0,2589	0,1535	1,6368
**Tc 26**	0,1337	0,1356	0,1034	0,2181	0,4861
**Tc 27**	0,3005	0,1835	0,1644	0,1039	1,4657
**Tc 28**	0,1585	0,0994	0,1058	0,0670	0,3029
**Tc 29**	0,2238	0,1345	0,0961	0,0218	0,3429
**Tc 30**	0,1289	0,1293	0,1014	0,0508	0,4500
**Tc 31**	0,2177	0,1239	0,1069	0,0297	0,4389
**Tc 32**	0,2095	0,0803	0,0745	0,0695	0,5403
**Tc 33**	0,2049	0,1216	0,0747	0,0314	0,4612
**Tc 34**	0,2140	0,1739	0,1715	0,0665	0,5414
**Tc 35**	0,2464	0,1518	0,1615	0,0872	0,3614
**Tc 36**	0,1814	0,1460	0,0732	0,1246	0,3852
**Tc 37**	0,5506	0,2304	0,1747	0,4897	0,6417
**Tc 38**	0,1662	0,2078	0,1915	0,1599	0,4463
**Tc 39**	0,2484	0,2044	0,3086	0,2351	0,6003
**Tc 40**	0,1665	0,2001	0,2204	0,0790	0,4601
**Tc 41**	0,4179	0,2896	0,2050	0,1647	1,2159
**Tc 42**	0,4672	0,2667	0,2560	0,4118	1,3925
**Tc 43**	0,5586	0,2747	0,0943	0,2568	1,1576
**Tc 44**	0,4654	0,1611	0,1878	0,9920	1,6182
**Tc 45**	0,3584	0,1507	0,1100	0,1525	1,3910
**Tc 46**	0,3997	0,2246	0,1788	0,6110	1,4857
**Tc 47**	0,6092	0,2069	0,2622	0,4807	1,2134
**Tc 48**	0,6864	0,2185	0,1571	0,3732	1,4390

Dogs infected with *Babesia* sp.					
**Antigens**	**KDDR-PLUS**	**KDDR**	**K39**	**K26**	**Crude Soluble Antigen**
**Cut-off**	**0,4087**	**0,3248**	**0,3147**	**0,3251**	**0,8314**
**ID**	**Absorbances**				
**Bab 01**	0,3533	0,1893	0,1888	0,1729	0,3637
**Bab 02**	0,4338	0,2313	0,1727	0,1872	0,4258
**Bab 03**	0,2511	0,2308	0,1287	0,2379	0,3488
**Bab 04**	0,1090	0,1424	0,1656	0,0337	0,3539
**Bab 05**	0,2436	0,1204	0,1525	0,1494	0,4499
**Bab 06**	0,2723	0,1106	0,0883	0,2385	0,4244
**Bab 07**	0,5750	0,1356	0,2410	0,7731	0,7472
**Bab 08**	0,3246	0,1379	0,3636	0,2186	0,5046
**Bab 09**	0,3608	0,1981	0,1356	0,5341	1,3814
**Bab 10**	0,7660	0,3323	0,2895	0,1618	1,3623
**Bab 11**	0,1859	0,6578	0,3127	0,2141	1,2988
**Bab 12**	0,0657	0,1527	0,0548	0,1000	1,0261
**Bab 13**	0,2695	0,5700	0,3338	0,0608	1,3235
**Bab 14**	0,1319	0,0674	0,0347	0,0856	1,1554
**Bab 15**	0,2200	0,2215	0,1101	0,3826	1,4270
**Bab 16**	0,4673	0,3327	0,2170	0,2383	1,4684
**Bab 17**	0,3747	0,2395	0,1455	0,6809	1,4587
**Bab 18**	0,1461	0,0816	0,0361	0,1256	1,2666
**Bab 19**	0,2896	0,2433	0,1616	0,5272	1,7907
**Bab 20**	0,4247	0,2926	0,1957	0,3391	1,4259
**Bab 21**	0,2963	0,3628	0,2511	0,3165	1,7810
**Bab 22**	0,0931	0,2250	0,0847	0,1465	1,2564
**Bab 23**	0,3837	0,4044	0,2097	0,1208	1,4283
**Bab 24**	0,2157	0,2974	0,1696	0,3228	1,2148
**Bab 25**	0,3731	0,1984	0,0828	0,1361	0,4195
**Bab 26**	0,3182	0,2998	0,3351	0,7420	0,9109
**Bab 27**	0,7603	0,2569	0,1207	0,2506	0,4535

Dogs infected with *Ehrlichia* sp.					
**Antigens**	**KDDR-PLUS**	**KDDR**	**K39**	**K26**	**Crude Soluble Antigen**
**Cut-off / ID**	**0,4087**	**0,3248**	**0,3147**	**0,3251**	**0,8314**
	**Absorbances**				
**Ehr 01**	0,8500	0,2847	0,2945	0,0920	1,0179
**Ehr 02**	0,1877	0,1983	0,1326	0,1247	0,8848
**Ehr 03**	0,2798	0,2790	0,1829	0,1991	1,4985
**Ehr 04**	0,2511	0,3374	0,2899	0,4531	1,6038
**Ehr 05**	0,3178	0,2551	0,2220	0,2724	1,1207
**Ehr 06**	0,6440	0,2571	0,2237	0,0499	0,5471
**Ehr 07**	0,3693	0,2205	0,1812	0,6464	0,9088
**Ehr 08**	0,1300	0,3327	0,1440	0,4282	0,8201
**Ehr 09**	0,2311	0,1179	0,1144	0,9719	0,8249
